# The Adjunctive Use of Leucocyte- and Platelet-Rich Fibrin in Periodontal Endosseous and Furcation Defects: A Systematic Review and Meta-Analysis

**DOI:** 10.3390/ma15062088

**Published:** 2022-03-11

**Authors:** Eudoxie Pepelassi, Maria Deligianni

**Affiliations:** 1 Department of Periodontology, School of Dentistry, National and Kapodistrian University of Athens, 115 27 Athens, Greece; 2 Bioinformatics and Computational Biology, School of Science, Department of Biology, National and Kapodistrian University of Athens, 157 01 Athens, Greece; mdeligianni@biol.uoa.gr

**Keywords:** endosseous defects, furcation defects, intrabony defects, L-PRF, platelet-rich fibrin, osseous regeneration, periodontal regeneration, periodontitis, meta-analysis, systematic review

## Abstract

The aim of this systematic review of randomized controlled trials was to evaluate the adjunctive use of leucocyte- and platelet-rich fibrin (L-PRF) in periodontal endosseous and furcation defects, as compared without L-PRF. The endosseous defect group was subclassified into: L-PRF/open flap debridement (L-PRF/OFD) versus OFD, L-PRF/osseous graft (L-PRF/OG) versus OG, L-PRF/Emdogain (L-PRF/EMD) versus EMD, and L-PRF/guided tissue regeneration (L-PRF/GTR) versus GTR. The furcation defect group was subclassified into L-PRF/OFD versus OFD, and L-PRF/OG versus OG. Mean difference, 95% confidence intervals and forest plots were calculated for probing pocket depth (PPD), clinical attachment level (CAL) and radiographic defect depth (DD). Nineteen studies concerning systemically healthy non-smokers were included. The results of this systematic review and meta-analysis showed in two- and/or three-wall endosseous defects that the adjunctive use of L-PRF to OFD or OG was significantly beneficial for PPD reduction, CAL gain and DD reduction, as compared without L-PRF. Furthermore, the data showed that for two- and/or three-wall endosseous defects, the adjunctive use of L-PRF to GTR was significantly beneficial for CAL and DD improvement, whereas adding L-PRF to EMD had no significant effect, and that for class II furcation defects, the addition of L-PRF to OFD was significantly beneficial for PPD, CAL and DD improvement, whereas the addition of L-PRF to OG was significantly clinically beneficial. In conclusion, this systematic review and meta-analysis found that there was significant clinical and radiographic additive effectiveness of L-PRF to OFD and to OG in two- and/or three-wall periodontal endosseous defects of systemically healthy non-smokers, as compared without L-PRF.

## 1. Introduction

Periodontal regeneration (or regeneration) is histologically defined as the regeneration of the tooth supporting tissues, which involves the alveolar bone, cementum and periodontal ligament, over a previously diseased root surface [1]. The surgical techniques currently used to regenerate the periodontal tissues include guided tissue regeneration (GTR) with barrier membranes (non-resorbable or resorbable), osseous grafts (OG), biologic mediators of regeneration and combination of more than one of these techniques. Enamel matrix derivative (EMD) is the mostly documented biologic mediator of regeneration. Their outcome is assessed clinically, radiographically and histologically. The regenerative techniques are applied in periodontal endosseous (or intrabony) defects and class II furcation defects. Regeneration of the periodontal tissues in human periodontal endosseous defects can be achieved to varying degrees by using various regenerative techniques [2]. Based on the most recent guidelines of the European Federation of Periodontology (EFP), EMD or GTR combined with papilla preservation flaps should be considered the treatment of choice for residual pockets with deep (≥3 mm) endosseous defects [3]. Research in periodontal regeneration is mainly focused on the combination of regenerative means and techniques that might enhance regeneration, such as the biologic mediators of regeneration. The new types of autologous platelet concentrates (APCs) are among the biologic mediators of regeneration being studied in endosseous and furcation defects. It is worthwhile mentioning that the use of PRF has been recently expanded to endodontic-periodontal defects with encouraging preliminary results [4]. In 2009, Dohan Ehrenfest et al. classified platelet concentrates into four categories based on their leucocyte and fibrin content: pure platelet-rich plasma (P-PRP), leucocyte-and platelet-rich plasma (L-PRP), pure platelet-rich fibrin (P-PRF), and leucocyte- and platelet-rich fibrin (L-PRF) [5]. They differ in preparation protocol, content, physical and biologic characteristics. Since then, advanced platelet-rich fibrin (A-PRF) [6], titanium-prepared platelet rich fibrin (T-PRF) [7], injectable platelet-rich fibrin (I-PRF) [8] and concentrated platelet-rich fibrin (C-PRF) [9] have been introduced. PRF is prepared by centrifugation of a blood sample at 3000 rpm for 10 min [10], which induces massive platelet activation [11] and not requiring anticoagulants or bovine thrombin. The content of PRF is mainly cytokines, glycanic chains and glycoproteins entrapped within a fibrin network [12]. L-PRF has high fibrin content and strong fibrin network with good mechanical properties [5], which were found superior to certain APCs [13]. L-PRF is used in clot form to fill the defect and in membrane form to cover it [14]. A-PRF is prepared by centrifugation of a blood sample at 1500 rpm for 14 min [6]. For A-PRF, platelets are allocated more evenly, and neutrophilic granulocytes are differently distributed [6].

**Figure 1:**
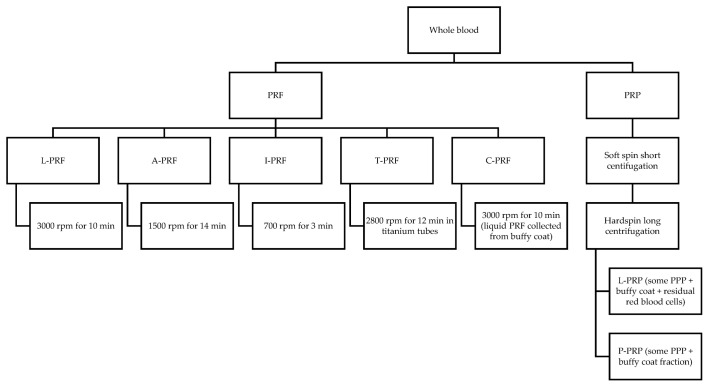
Preparation protocols of APCs. Abbreviations: PRF: Platelet-rich fibrin; L-PRF: leucocyte- and platelet-rich fibrin; A-PRF: advanced platelet-rich fibrin; I-PRF: injectable platelet-rich fibrin; T-PRF: titanium-prepared platelet-rich fibrin; C-PRF: concentrated platelet-rich fibrin; PRP: platelet-rich plasma; L-PRP: leucocyte-platelet-rich plasma; PPP: platelet-poor plasma; P-PRP: pure platelet rich plasma; rpm: rounds per minute, min: minutes.

Growth factor release kinetics and concentration differ among PRP, PRF and A-PRF. For PRP, the release of growth factors starts earlier [15]. For PRF, the release of growth factors is longer, with a steady release for at least 7 days [11,16] or even 10 days [15]. For A-PRF, the release is quantitatively higher as compared with PRF [15]. Based on the hypothesis that titanium might be more effective in platelet activation, T-PRF was produced, which was prepared by using titanium tubes centrifuged at 2800 rpm for 12 min [7]. Fibrin is thicker and tighter for T-PRF than L-PRF [7]. T-PRF, as compared with L-PRF, has significantly more T cells, B-lymphocytes and platelets with a strongly positive immunohistochemical staining in terms of cell distribution and labeling index. In terms of localization, for T-PRF there is a stronger positive staining with platelets, whereas for L-PRF there is a stronger positive staining with stem cells. In terms of cell pattern, for T-PRF there is a significantly stronger positive staining with B-lymphocytes, whereas for L-PRF there is a significantly stronger positive staining with neutrophils [17]. I-PRF is prepared by centrifugation of a blood sample at 700 rpm for 3 min [8]. Anti-inflammatory effect has been reported for I-PRF [18] and L-PRF [19]. C-PRF is the liquid PRF that is directly collected from the buffy coat layer (directly above the red blood layer) after standard L-PRF preparation [9]. C-PRF as compared with I-PRF has statistically significant increase in leukocyte (>500%) and platelet (>1500%) counts [9], growth factor release and gingival fibroblast migration [20]. Preparation protocols of autologous platelet concentrates are shown in Figure 1.

The use of PRF in endosseous and furcation defects is steadily increasing in the attempt to improve the outcomes of the periodontal treatment. In terms of the use of PRF in endosseous and furcation defects, central questions are:

✓ “Is the addition of PRF to other surgical techniques beneficial?”;
✓ “Is the addition of PRF to other regenerative techniques, such as osseous grafts, GTR or EMD, beneficial?”.

The aim of the present systematic review was to evaluate the adjunctive use of L-PRF in the surgical treatment of periodontal endosseous and furcation defects, as compared without L-PRF.

## 2. Materials and Methods

### 2.1. Protocol

The present study was conducted based on the guidelines of the “Cochrane Handbook for Systematic Reviews of Interventions” [21] and is reported following the “Preferred Reporting Project Guidelines for Systematic Review and Meta-analysis” (PRISMA) statement.

### 2.2. Eligibility Criteria

The inclusion criteria were based on the PICOS strategy. Studies not meeting the following criteria were excluded.

#### 2.2.1. Types of Participants

Systemically healthy individuals, regardless of age or gender, presenting chronic periodontitis and periodontal endosseous or furcation defects requiring surgical intervention were included in the study. Studies including smokers were excluded.

#### 2.2.2. Types of Interventions

Surgical treatment of endosseous or furcation defects with the use of L-PRF alone or in combination with other biomaterials. A follow-up period of at least 6 months was required.

#### 2.2.3. Type of Comparison

L-PRF versus open flap debridement alone or in combination with other biomaterials. The experimental intervention was L-PRF used as an adjunct to other surgical techniques, specifically to open flap debridement (OFD), OG, GTR and EMD. The control intervention was the same surgical technique when used without the adjunct of L-PRF.

#### 2.2.4. Type of Outcome Measures

The primary measures were the change in probing pocket depth (PPD), clinical attachment level (CAL) and radiographic depth of the endosseous defect (defect depth, DD). Specifically, the PPD reduction, CAL gain and defect depth reduction (DD reduction). The secondary measures were the change in gingival margin level (GML), the radiographic fill of the endosseous defect (defect fill, DF), expressed as percentage (%), and the wound healing.

#### 2.2.5. Types of Studies

Randomized controlled trials (RCTs), both of parallel and split-mouth design, were included in the study. Controlled trials and studies with duration <6 months were excluded.

### 2.3. Search Strategy

Searches were conducted in the following databases for RCTs and controlled trials, without language, publication year and publication status restrictions.

- PubMed (searched 16 June 2021) (Listing S1);
- Scopus (searched 16 June 2021) (Listing S2);
- Cochrane Library (searched 16 June 2021) (Listing S3);
- Lilacs (searched 16 June 2021) (Listing S4);
- Grey Literature Report (searched 16 June 2021) (Listing S5).

### 2.4. Selection Process

Following the electronic search, the titles and abstracts were screened to exclude all articles not meeting the inclusion criteria. Trials that were not randomized were excluded. Of the remaining articles, full texts were acquired and assessed. Studies that did not fully meet the inclusion criteria were excluded.

### 2.5. Data Synthesis

Both authors reviewed the included studies and independently extracted the data. The extracted information was: (a) first author name, publication year; (b) study design; (c) patient characteristics, namely, gender, age, inclusion and exclusion criteria; (d) comparisons, PRF preparation protocol, and surgical technique; and (e) outcomes, such as PPD, CAL, GML and DD.

### 2.6. Risk of Bias Assessment

The risk of bias was assessed according to the “Cochrane Handbook for Systematic Reviews of Interventions” [22] using the RoB 2 tool for risk of bias assessment. Each study was analyzed regarding five domains: risk of bias occurring from the randomization process and risk of bias due to allocation concealment (selection bias), risk of bias in relation to blinding the participants and the personnel (performance bias), risk of bias in the measurement of outcomes (detection bias), risk of bias due to missing outcome data (attrition bias) and risk of bias in the selection of reported data (reporting bias). Overall risk of bias was assessed according to the guidelines: if all five domains were at low risk, overall risk of bias was low. If at least one domain was at unclear risk, then overall risk was deemed as unclear risk. Finally, if at least one domain was at high risk of bias, then overall risk was assessed as high risk. These assessments are reported both in Table 1 and graphically (Figure S1).

### 2.7. Data Analysis

The continuous variables (PPD, CAL, DD) were categorized in groups and subgroups and analyzed using Review Manager software (Review Manager (RevMan) computer program, Version 5.4, The Cochrane Collaboration, 2020). Estimates of the effect sizes were expressed as mean difference (MD) with 95% confidence intervals (CI). The inverse variance method was used for fixed effects or random effects, depending on the heterogeneity between studies. Heterogeneity was assessed using the Chi^2^ test. Values of I^2^ ≤ 25% were deemed as low heterogeneity, while values >25% and <50% were classified as moderate, and values ≥50% were classified as high heterogeneity. A random effects model was used when heterogeneity was found to be high. The statistical significance level of the effect of this meta-analysis was fixed at *p* < 0.05.

## 3. Results

### 3.1. Study Selection

**Figure 2:**
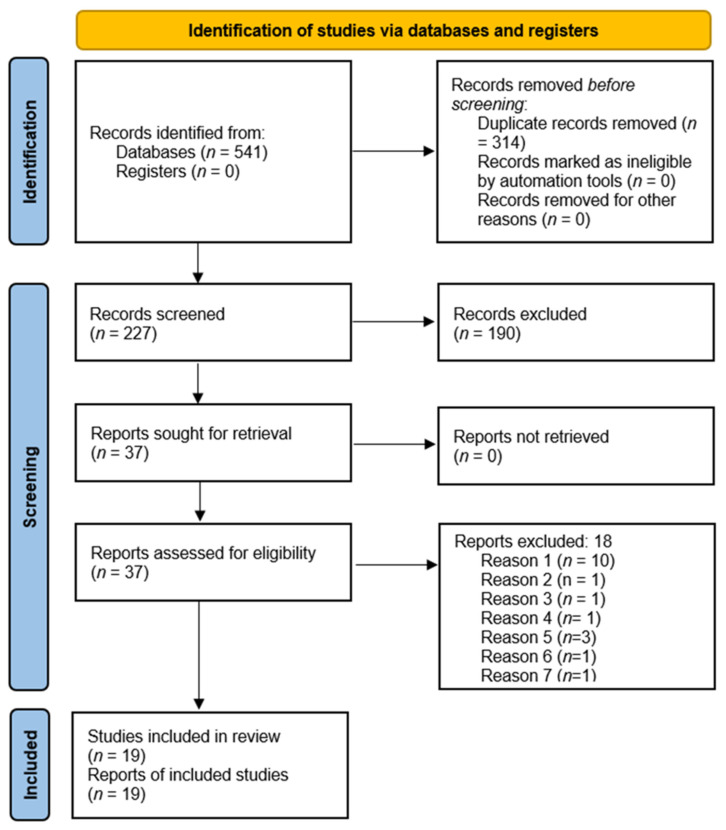
PRISMA study flow diagram.

A total of 541 articles were identified through electronic search and none were obtained through other sources. After duplicates were removed, 230 records were screened by reading titles and abstracts and 190 articles were excluded for not meeting the inclusion criteria (patients with aggressive periodontitis, controlled clinical trials, study duration less than 6 months and studies not using PRF as an adjunct). Thirty-seven articles were carefully read and 19 of them met the inclusion criteria and were selected for qualitative and quantitative analysis. The study selection is depicted in the PRISMA flow diagram in Figure 2. The 18 studies excluded after being assessed for eligibility are displayed in Table 1.

### 3.2. Study Characteristics

The characteristics of the included studies are displayed in Table 2. All 19 studies were RCTs, of which 9 were parallel (7 for endosseous and 2 for furcation defects) and 10 were split-mouth (9 for endosseous and 1 for furcation defects). All individuals were systemically healthy, non-smokers presenting chronic periodontitis. All studies except one, treated two- and/or three-wall endosseous or class II furcation defects. For all studies, initial periodontal therapy was performed before the surgical intervention. All patients maintained proper oral hygiene during the follow-up period of 6–12 months. Of the 19 studies, 12 used the 3000 rpm for 10-min protocol (63% of studies), four used the 2700 rpm for 12 min protocol (21% of studies), two used the 3000 rpm for 12 min protocol (11% of studies) and one used the 2700 rpm for 10 min protocol (5% of studies).

### 3.3. Risk of Bias in Individual Studies

**Figure 3:**
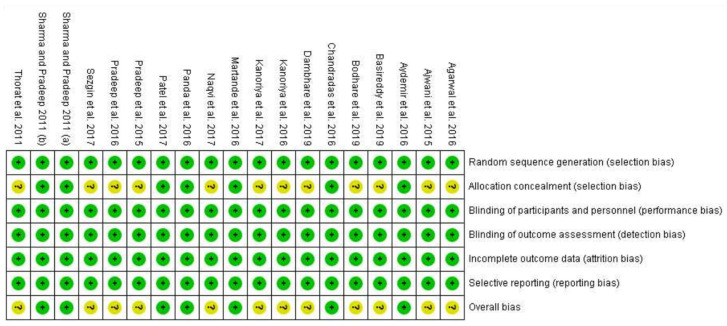
Risk of bias summary. 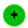: Low Risk. 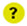: Unclear Risk.

**Figure 4:**
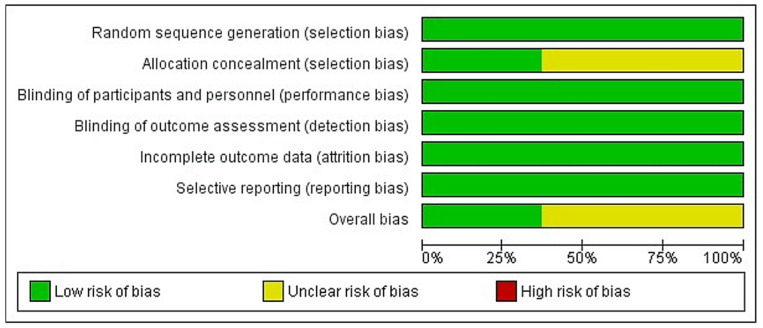
Risk of bias shown as a percentage (%).

The risk of bias is presented as a percentage across all included studies in Figure 3, and the individual studies are shown in Figure 4. Randomization of participants was achieved in all studies either with computer generated list, toss of a coin or draw of chits. Allocation concealment was reported in nine of 19 studies, using opaque envelopes. Regarding blinding, examiners were blinded in all studies, however due to the nature of the treatment, operators could not be blinded. Finally, most individuals completed the studies and the reporting of data was appropriate in all studies.

### 3.4. Synthesis of Results

In order to analyze the data, six subgroups (four for endosseous defects, and two for furcation defects) were created, as follows:

- For endosseous defects:
  ° (L-PRF + OFD) vs. OFD alone;
  ° (L-PRF + OG) vs. OG alone;
  ° (L-PRF + EMD) vs. EMD alone;
  ° (L-PRF + GTR) vs. GTR alone.
- For furcation defects:
  ° ° (L-PRF + OFD) vs. OFD alone;
  ° ° (L-PRF + OG) vs. OG alone.

**Figure 5:**
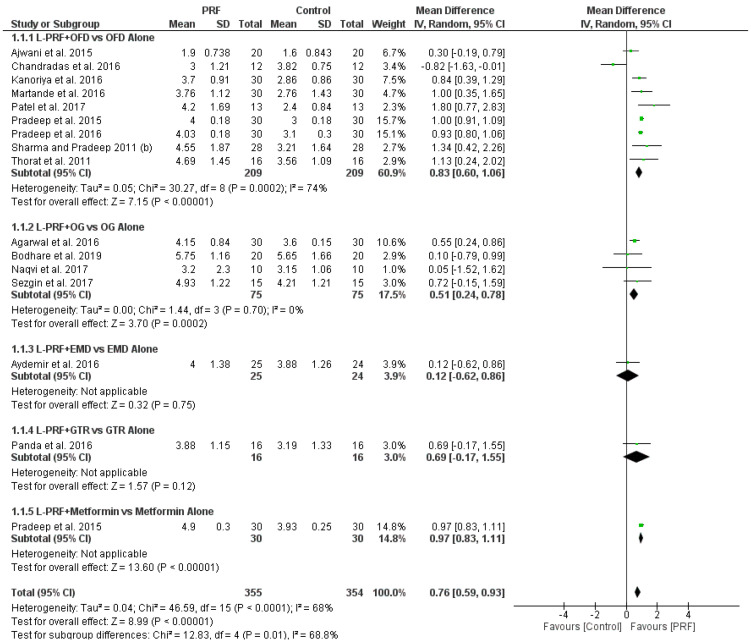
Forest plot of comparison. 1. Endosseous defects, outcome; 1.1. Probing pocket depth reduction.

Meta-analysis was performed in two endosseous defects groups, namely, (L-PRF + OFD) vs. OFD alone, and (L-PRF + OG) vs. OG alone. The forest plots of comparisons for endosseous and furcation defects are shown in Figure 5 as well as in Supplementary Materials (Figures S2–S6).

#### 3.4.1. (L-PRF + OFD) vs. OFD Alone in Endosseous Defects

Nine out of 19 RCTs compared the effectiveness of L-PRF following OFD to that of OFD alone. Regarding clinical parameters, the addition of L-PRF to OFD led to statistically significant differences in PPD reduction in seven out of nine studies with a mean difference of 0.83 mm with 95% probability that the true mean PPD reduction estimate is between 0.6 and 1.6 mm, (high heterogeneity (I^2^ = 74%) random effects model used, mean difference 0.83 mm, 95% CI: 0.60 to 1.60), favoring L-PRF, and to statistically significant differences in CAL gain in seven out of nine studies with a mean difference of 1.02 mm and 95% probability that the true mean CAL gain is between 0.83 and 1.21 mm (high heterogeneity (I^2^ = 55%) random effects model used, mean difference 1.02 mm, 95% CI: 0.83 to 1.21) favoring L-PRF. In terms of radiographic parameters, the addition of L-PRF to OFD led to statistically significant differences in DD reduction in seven out of eight studies (one study did not record DD reduction) with an estimated mean DD reduction of 1.82 mm (high heterogeneity (I^2^ = 87%) random effects model used, mean difference 1.82 mm, 95% CI: 1.59 to 2.05) indicating an advantage in using L-PRF.

#### 3.4.2. (L-PRF + OG) vs. OG Alone in Endosseous Defects

Four out of 19 RCTs compared the use of L-PRF as an adjunct to OG, to the use of OG alone. All four were split-mouth trials. In terms of CAL gain, the addition of L-PRF to OG led to statistically significant differences in three out of four studies, with mean CAL gain of 1.08 mm (low heterogeneity (I^2^ = 0%) fixed effects model used, mean difference 1.08 mm, 95% CI: 0.78 to 1.39) favoring the combined L-PRF/OG approach. In terms of PPD reduction, the addition of L-PRF to OG led to statistically significant difference in one out of four studies (low heterogeneity (I^2^ = 0%) fixed effects model used, mean difference 0.51 mm, 95% CI: 0.24 to 0.78). In terms of radiographic parameters, the addition of L-PRF to OG led to statistically significant differences in DD reduction in three out of four studies, with 95% probability that the true mean DD reduction is between 0.66 and 1.20 mm (low heterogeneity (I^2^ = 0%) fixed effects model used, mean difference 0.93 mm, 95% CI: 0.66 to 1.20) indicating an advantage using L-PRF as an adjunct to OG.

#### 3.4.3. (L-PRF + EMD) vs. EMD Alone in Endosseous Defects

One study evaluated the adjunctive use of L-PRF to EMD, as compared with EMD alone, where there were no statistically significant differences between test and control groups. In the meta-analysis, a small non-statistically significant advantage in using L-PRF was found regarding PPD (mean difference: 0.12 mm, 95% CI: −0.62 to 0.86) and CAL (mean difference: 0.13 mm, 95% CI: −0.59 to 0.85), while DD reduction showed a small non-statistically significant disadvantage in adding L-PRF to EMD (mean difference: −0.02 mm, 95% CI: −0.62 to 0.58). Nevertheless, more studies need to be conducted comparing the combination of L-PRF and EMD, to EMD alone, in order to address the efficacy of L-PRF.

#### 3.4.4. (L-PRF + GTR) vs. GTR Alone in Endosseous Defects

One study evaluated the use of L-PRF as filler in combination with GTR, as compared with GTR alone, where statistically significant differences were found in terms of CAL gain and DD reduction. The meta-analysis found a statistically significant advantage in using L-PRF regarding CAL gain (mean difference: 1.06, 95% CI: 0.04 to 2.08) and DD reduction (mean difference: 1.30 mm, 95% CI: 0.96 to 1.64) and a non-statistically significant advantage in using L-PRF regarding PPD reduction (mean difference: 0.69 mm, 95% CI: −0.17 to 1.55). However, more studies need to be conducted comparing the combined use of L-PRF and GTR, to GTR alone, in order to address the efficacy of L-PRF.

#### 3.4.5. (L-PRF + Metformin) vs. Metformin Alone in Endosseous Defects

One study evaluated the adjunctive use of L-PRF to metformin, as compared with metformin alone, with statistically significant differences between groups. In the meta-analysis, statistically significant advantage in using L-PRF was found regarding PPD (mean difference: 0.97 mm, 95% CI: 0.83 to 1.11), CAL (mean difference: 0.97 mm, 95% CI: 0.83 to 1.11) and DD (mean difference: 0.21 mm, 95% CI: 0.06 to 0.36). However, more studies need to be conducted comparing the combination of L-PRF and metformin, to metformin alone, in order to address the efficacy of L-PRF.

#### 3.4.6. (L-PRF + OFD) vs. OFD Alone in Furcation Defects

Two studies evaluated the effectiveness of L-PRF following OFD, to that of OFD alone. Regarding PD reduction and CAL gain, statistically significant difference was found favoring the L-PRF group (mean difference: 1.20, 95% CI: 0.97, 1.42) and (mean difference: 1.06, 95% CI: 0.88, 1.23). In terms of DD reduction, the use of L-PRF led to a statistically significant improvement with mean difference of 1.56 mm and 95% CI: 1.48, 1.63. However, due to the low number of RCTs it must be noted that more studies need to be conducted in order to address the efficacy of L-PRF in the treatment of furcation defects.

#### 3.4.7. (L-PRF + OG) vs. OG Alone in Furcation Defects

Two studies evaluated the adjunctive use of L-PRF to OG, to the use of OG alone. No radiographic evidence was provided. In terms of PD reduction and CAL gain, statistically significant differences were found with mean difference and 95% CI: 0.64 mm (0.34, 0.94) and 0.84 mm (0.44, 1.23) respectively. More studies need to be conducted providing radiographic evidence and allowing further analysis of the data in order to evaluate whether L-PRF is effective as an adjunct to OG in furcation defects.

## 4. Discussion

The present systematic review and meta-analysis evaluated the adjunctive use of L-PRF in the surgical treatment of two- and/or three-wall endosseous defects and class II furcation defects, as compared without L-PRF, in 19 RCTs concerning systemically healthy non-smoking periodontitis patients. Concerning endosseous defects, most of the 16 studies compared either the use of L-PRF following OFD to OFD alone, or the combined use of L-PRF and OG to OG alone, whereas few studies evaluated the adjunctive use of L-PRF to GTR or EMD or metformin, as compared without L-PRF. Concerning furcation defects, the studies evaluated the adjunctive use of L-PRF to either OFD or OG, as compared without L-PRF.

### 4.1. Meta-Analysis

#### 4.1.1. (L-PRF + OFD) vs. OFD Alone in Endosseous Defects

Concerning endosseous defects, nine studies compared the combination of L-PRF and OFD to OFD alone. Seven out of these nine studies found a significant difference in PPD reduction and CAL gain favoring the L-PRF group. Eight out of eight studies (DD was not evaluated in one of the nine studies) found a significant difference in DD reduction favoring the L-PRF group. The present results support that the addition of L-PRF to OFD in two- and/or three-wall endosseous defects of systemically healthy non-smoking periodontitis patients is statistically significantly beneficial for PPD reduction, CAL gain and DD reduction, as compared with OFD alone. L-PRF has significant positive additional clinical and radiographic effect to OFD in two- and/or three-wall endosseous defects. L-PRF is significantly superior to OFD alone in terms of PPD reduction, CAL gain and DD reduction of endosseous defects. Concerning two- and/or three-wall endosseous defects, L-PRF is statistically significantly superior to OFD alone in terms of PPD reduction, CAL gain and DD reduction. For all studies (for nine out of nine studies), the risk of bias was not high, which should be stressed.

The results of the present study on endosseous defects can be compared with those of previous systematic reviews and meta-analyses [60,61,62,63,64]. The present clinical findings on L-PRF agree with the findings of Del Fabbro et al.’s [60] systematic review, where superiority of APCs in total over OFD was showed in endosseous defects. The present significant additive effectiveness of L-PRF to OFD in terms of PPD reduction, CAL gain and DD reduction is in agreement with the results of the systematic reviews and meta-analyses on PRF by Li et al. [62] and Chen et al. [64]. Furthermore, the present significant clinical (PPD, CAL) superiority of L-PRF over OFD agrees with the results of the systematic reviews and meta-analyses by Castro et al. [61] on L-PRF and Miron et al. [63] on PRF. The additive effectiveness of L-PRF to OFD in terms of CAL gain was slightly lower for the present study (1.02 mm (95% CI: 0.83 to 1.21)) than for the studies by Castro et al. [65] (1.2 ± 0.6 mm), Chen et al. [64] (1.25 mm (95% CI: 0.93 to 1.57)) and Miron et al. [63] (1.39 mm (95% CI: 1.03 to 1.76)). The further PPD reduction achieved with the combined treatment was slightly lower in the present study (0.83 mm (95% CI: 0.60 to 1.06)) than in the studies by Castro et al. [65] (1.1± 0.5 mm) and Miron et al. [63] (1.26 mm (95% CI: 0.99 to 1.53)). In terms of DD reduction, the present study (1.82 mm (95% CI: 1.59 to 2.05)) and Chen et al.’s [64] (1.81 mm (95% CI: 1.53 to 2.08)) systematic review and meta-analysis shared the same mean additive effectiveness of PRF to OFD.

In the present study, the additive clinical effectiveness of L-PRF to OFD is statistically significant, though the arithmetic mean differences for PPD reduction (0.83 mm (95% CI: 0.60 to 1.06) and CAL gain (1.02 mm (95% CI: 0.83 to 1.21) are small, which might arise questions on the clinical significance of adding L-PRF to OFD. In the present study, with the combined L-PRF/OFD approach (as compared with OFD alone) the mean difference in DD reduction (1.82 mm (95% CI: 1.59 to 2.05) is higher than the mean difference in CAL gain (1.02 mm (95% CI: 0.83 to 1.21). The higher mean difference in DD reduction than in CAL gain found in the present study with the combined L-PRF/OFD, as compared with OFD alone, is in accordance with findings in the systematic review and meta-analysis by Chen et al. [64]. The statistically significant further DD reduction achieved in this study by adding L-PRF to OFD (1.82 mm (95% CI: 1.59 to 2.05)) seems to be clinically significant as well. Further reducing the endosseous DD by almost 2 mm might prove to be more important than improving PPD and CAL by 1 mm. In this context, it seems that the addition of L-PRF to OFD in endosseous defects is more justified for the radiographic improvement expected to be achieved, than for the clinical one. It could be suggested that expectations are higher for radiographic than clinical improvement when adding L-PRF to OFD in endosseous defects.

#### 4.1.2. (L-PRF + OG) vs. OG Alone in Endosseous Defects

Four studies compared L-PRF as an adjunct to OG in endosseous defects. One out of these four studies found statistically significant difference in PPD reduction, and three out of four studies found statistically significant difference in CAL gain favoring the L-PRF group. Three out of four studies found a significant difference in DD reduction favoring the L-PRF group. The present results support that the addition of L-PRF to OG in two- and/or three-wall endosseous defects of systemically healthy non-smoking chronic periodontitis patients is statistically significantly beneficial for PPD reduction, CAL gain and DD reduction. Thus, for two- and three-wall endosseous defects it seems that adding L-PRF to an osseous graft might be justified regarding the clinical and radiographic improvement. Though there is statistical significance of the additive effectiveness of L-PRF to OG in terms of PPD reduction and CAL gain, its clinical significance is questioned due to the small arithmetic difference (0.51 mm and 1.08 mm, respectively). Similarly, the additive effectiveness of L-PRF to OG in terms of DD reduction is relatively small (0.93 mm). The present results can be compared with those of two recent systematic reviews and meta-analyses of RCTs in two- and three-wall endosseous defects of systemically healthy patients that explored clinically and radiographically the possible additional effect of PRF to osseous grafts [63,64]. The present significant additive effectiveness of L-PRF to OG in terms of PPD reduction and DD reduction is in agreement with the findings by Chen et al. [64] in non-smokers. The present statistically significant additive effectiveness of L-PRF to OG in terms of CAL gain is in agreement with the findings by Chen et al. [64] and Miron et al. [63]. Interestingly, the mean differences in CAL gain (1.08 mm vs. 1.09 mm) and DD reduction (0.93 mm vs. 0.92 mm) were almost the same for the present and Chen et al.’s study [64].

### 4.2. Additional RCTs Evaluating L-PRF in Endosseous Defects

There are additional studies comparing the addition of L-PRF to other treatment modalities in endosseous defects [44,54,55], where no meta-analysis could be performed, as follows.

#### 4.2.1. (L-PRF + EMD) vs. EMD Alone in Endosseous Defects

The combination of L-PRF and EMD in one-, two-, three-wall endosseous defects of non-smoking chronic periodontitis patients was compared with EMD alone in one study [55], where the results were similar for both groups in terms of PPD, CAL and DD improvement.

#### 4.2.2. (L-PRF + GTR) vs. GTR Alone in Endosseous Defects

One study evaluated the combination of L-PRF and GTR in three-wall endosseous defects of non-smoking chronic periodontitis patients, as compared with GTR alone, and found significantly higher CAL gain and DD reduction for the combined treatment approach [54].

#### 4.2.3. (L-PRF + Metformin) vs. Metformin Alone in Endosseous Defects

Lately, several studies addressed the use of L-PRF in combination with biomolecules, such as metformin, atorvastatin, rosuvastatin and bisphosphonates [44,45,47,48,57]. In these studies, L-PRF was used as a three-dimensional matrix acting as a drug delivery system, indicating the possibility of creating more personalized treatment protocols with the adjunctive use of L-PRF.

In the present study, the combined use of L-PRF to such a biomolecule, as compared with the biomolecule alone, was evaluated for metformin in one RCT. The addition of L-PRF to metformin significantly improved the results achieved with metformin alone in terms of PPD, CAL and DD. The preliminary findings on the use of L-PRF as a drug delivery system seem promising. Future research might enlighten the application of L-PRF as a drug delivery system concerning efficacy and mode of use.

### 4.3. Furcation Defects

#### 4.3.1. (L-PRF+ OFD) vs. OFD Alone in Furcation Defects

Two studies evaluated the adjunctive use of L-PRF to OFD in furcation defects. Both studies found significant difference in PPD reduction, vertical and horizontal CAL gain and DD reduction favoring the L-PRF group. Concerning furcation defects, the present results revealed significant additive effectiveness of L-PRF to OFD in terms of PPD reduction (1.20 mm), vertical and horizontal CAL gain (1.06 mm) and DD reduction (1.72 mm). The present results on significant additive effectiveness of L-PRF to OFD in furcation defects agree with previous systematic reviews on L-PRF [61] and PRF [66,67]. Specifically, the present significant clinical (in terms of PPD reduction and CAL gain) superiority of the combined L-PRF/OFD over OFD in furcation defects is in agreement with the results of the systematic reviews and meta-analyses by Castro et al. [61] on L-PRF, Tarallo et al. [67] on PRF and Panda et al. [66] on APCs (PRP and PRF in total). The additional improvement in PPD (1.20 mm vs. 1.9 mm) and CAL (1.06 mm vs. 1.32 mm) with the addition of L-PRF to OFD was similar for the present study and the systematic review by Castro et al. [61].

#### 4.3.2. (L-PRF+ OG) vs. OG Alone in Furcation Defects

Two studies evaluated the adjunctive use of L-PRF to OG in furcation defects. One out of two studies found significant difference in PPD reduction favoring the L-PRF group. One study found significant difference in CAL gain, whereas the other study found significant difference in horizontal CAL gain only (not in vertical), favoring L-PRF. DD reduction was evaluated in one of the two studies and it was found similar for both groups. For furcation defects, the present results revealed significant additive effectiveness of L-PRF to OG in terms of PPD reduction (0.64 mm) and CAL gain (0.84 mm). The present results on additive effectiveness of L-PRF in CAL gain are in agreement with findings by Tarallo et al. [67] and Panda et al. [66].

### 4.4. Secondary Outcomes

#### 4.4.1. Gingival Margin Level (GML) Change

Ten out of 19 studies showed statistically significant difference in GML change favoring the L-PRF group, while five studies did not record this parameter, and four studies did not find significant differences between groups.

#### 4.4.2. Percentage Defect Fill (%DF)

Ten out of 19 studies showed statistically significant difference in percentage DF favoring the L-PRF group, while six studies did not record this parameter and three studies did not find significant differences between groups.

#### 4.4.3. Uneventful Wound Healing

Only one study recorded early wound healing [49] showing statistically significant difference favoring the L-PRF group. The remaining studies reported uneventful wound healing in all cases, showing the wound healing properties of L-PRF.

### 4.5. Future Research Directions

All the studies included in the present systematic review and meta-analysis evaluated two- and/or three-wall endosseous defects, except for the L-PRF/EMD study that evaluated one- and/or two- wall studies additionally, and analyzed the defects separately based on their configuration [53]. The present study found that in two- and/or three-wall endosseous defects the addition of PRF to OFD improves the clinical and radiographic outcome achieved with the sole use of OFD. Two- and/or three-wall endosseous defects are contained (or space maintaining) defects with high regenerative potential. Defect characteristics in terms of extent, osseous wall number, radiographic angulation and space maintenance need should play a role in the decision to use L-PRF following OFD. At the present time, PRF as sole grafting material might be considered for two- and/or three-wall endosseous defects. Using PRF following OFD in one- and two-wall (non-contained) endosseous defects is not justified. L-PRF clots alone are too difficult to stay in place in non-contained defects, due to their physical characteristics. In case of regenerative attempt in non-contained defects, another regenerative approach or the combination of PRF to other regenerative techniques might be selected.

Among all new types of APCs, L-PRF is one of the most widely documented. Significant superiority of any type has not been documented in endosseous and class II furcation defects. We assume that all new APCs act in a similar way to L-PRF. However, conclusions on each of them cannot be drawn without testing them separately. Based on their differences in content, physical and biologic characteristics in addition to growth factor release kinetics and concentration, differences in effectiveness cannot be ruled out. Selecting a specific APC among all APC types might prove to be important for defects with reduced regenerative potential, such as non-contained defects. Therefore, comparisons among the APC types in various types of endosseous and furcation defects should be made in properly designed RCTs. Moreover, most APCs should be compared with other regenerative techniques, and most APCs should be studied in combination with other regenerative techniques. Research should also focus on new types of PRF, such as T-PRF [7], A-PRF [6] and C-PRF [9], which were relatively recently introduced in RCTs. Preliminary findings indicate that the combined T-PRF/OFD might be superior to OFD in endosseous defects [68].

It should be stressed that there is no standard protocol for the preparation of L-PRF and for the clinical application of L-PRF in periodontal defects, which is a limitation. Concerning the preparation of L-PRF, there is variability in the literature since several centrifugation protocols have been described. Depending on the study, L-PRF has been used as filler or membrane (or cover) or both. The number of L-PRF clots to fill the endosseous or furcation defect and the number of L-PRF membranes to cover it varies among studies. In the present study, two similar L-PRF centrifugation protocols (3000 rpm × 10 min or 2700 rpm × 12 min) were included. All RCTs followed either one of these two protocols, which are widely accepted. In the present study, most RCTs on combined L-PRF/OFD in endosseous defects used both L-PRF clots and membranes, except for three trials where clots were used. Similarly, three out of four L-PRF/OG trials on endosseous defects followed the combined clot/membrane approach, whereas the fourth trial covered the defect with L-PRF membrane. Such variations were seen in the furcation trials included in this study, predominantly with the combined clot/membrane approach. Standardization of the L-PRF preparation protocol and of the L-PRF application mode per periodontal defect would help comparisons.

The present study included both parallel and split-mouth RCTs without separate sub-group analysis, based on the findings by Smaïl-Faugeron et al. [69]. Numerous systematic reviews and meta-analyses have included both split-mouth design and parallel-arm design studies, without separate sub-group analysis, in order to draw combined intervention effects. It has been suggested that the inclusion of studies of both split-mouth and parallel design in the same systematic review and meta-analysis without separate sub-group analysis entails the risk of negative effect on the outcomes [70], due to factors concerning the split-mouth design, such as carry-across effects (treatment performed in one oral site can affect the treatment response in other oral sites) [71], time period effects (time span between the first and second intervention sites), and statistical analysis methods (for paired and for non-paired sites). Smaïl-Faugeron et al. [69] conducted a meta-epidemiological study aiming to assess if data from split-mouth RCTs were incorporated appropriately in meta-analyses and whether intervention effect estimates differ between split-mouth and parallel-arm RCTs investigating the same questions in meta-analyses. Their study did not provide sufficient evidence for a difference in intervention effect estimates derived from split-mouth and parallel-arm RCTs and they suggested that authors should consider including split-mouth RCTs in their meta-analyses with suitable and appropriate analysis [69].

All RCTs included in this study evaluated non-smokers, which is a strength of the study. Smokers respond less favorably than non-smokers to periodontal flap surgical procedures [72] and to periodontal regeneration in endosseous defects [73]. Based on the consensus report from the 2015 American Academy of Periodontology (AAP) Regeneration Workshop, smoking negatively affects the efficiency of the regenerative techniques in periodontal endosseous defects, since a systematic review and meta-analysis aiming at evaluating the impact of smoking on osseous regeneration showed that in 60% of the studies, smoking statistically significantly negatively affected the post-operative defect fill [73].

The range of the postoperative follow-up time of the RCTs included in the present study was relatively small. Among all RCTs included in the present study, all trials lasted for equal to or more than 9 months, except for three where the duration was six months. All L-PRF/OFD studies lasted 9 months, except for a 12 month study. Long-term trials are required to properly assess L-PRF efficiency.

There are numerous RCTs on L-PRF use in endosseous defects, though histologic studies are lacking. Undoubtedly, “true” periodontal regeneration can be evaluated only by histology. Histologic evidence for periodontal regeneration is not yet available for PRP and PRF [2]. We assume that the radiographic defect fill achieved with PRF alone is bone fill, though it remains to be histologically proved. Histologic data on PRF exist for other types of osseous defects, such as experimental [74,75] and human alveolar ridge [76,77,78,79,80,81,82] defects. The combined PRF/β-TCP graft achieved more new bone formation at twelve weeks than PRF or β-TCP alone, as histologically assessed in experimental tibial defects in pigs [74]. L-PRF, bovine derived deproteinized xenograft (DBBM) and combined L-PRF/DBBM covered with collagen membranes had similar outcomes at nine weeks regarding percentage vital bone, percentage connective tissue and percentage remaining graft particles, as histologically assessed in experimental tibial defects in rabbits [75]. Regarding lateral two-stage sinus augmentation, the predominant human histologic data on the combined PRF/osseous graft, as compared with osseous graft alone, show that the amount of new bone formed is not affected and the bone formation process is accelerated leading to mature bone earlier [83]. Specifically, the combined PRF/osseous graft and the osseous graft alone were histologically similarly effective in lateral two-stage sinus augmentation in all the studies examined [77,79,80,81]. Furthermore, for the combined sinus graft there was non-statistically significantly longer contact length between new bone and bone substitute [77] and statistically significantly lower percentage of residual bone substitute [77]. Finally, accelerated healing of the bone formation process was histologically found for the combined sinus graft in two randomized controlled trials [78,82] and one retrospective study [84]. Certainly, direct comparisons of the healing process between periodontal endosseous defects and alveolar ridge defects cannot be made, though the above findings provide information on the PRF-mediated bone healing process in general. It should be stressed that sampling for histologic examination in augmented alveolar ridge sites is usually performed during drilling for implant placement. On the contrary, most studies in human periodontal defects evaluate the regenerative outcome clinically and radiographically but not histologically. Concerning regenerated periodontal defects, histologic evaluation is almost completely restricted to animal studies.

Further research is required on the comparison between L-PRF and standard periodontal regenerative procedures. Data on comparisons between L-PRF and other regenerative techniques are insufficient for endosseous defects and lacking for furcation defects. L-PRF membrane as an alternative to collagen membrane has not been tested in endosseous or furcation defects. Based on differences in physical characteristics and resorption time between collagen and PRF membranes [12,14], different responses might be anticipated when used as barriers in periodontal defects. PRF, as potent material for a barrier membrane, is easily manipulated and enhances healing. However, scaffolding and space maintenance effects are questioned. Using several layers of PRF membranes (usually compressed in one membrane) might affect their behavior in terms of strengthening and resorption delay. PRF and collagen membranes have been compared in maxillary sinus augmentation for covering the human lateral osteotomy site [84] and the perforated rabbit sinus membrane [85]. Covering the lateral osteotomy site with PRF or collagen membrane in the autograft/DBBM-mediated sinus augmentation did not statistically significantly affect the outcome regarding vital bone formation and residual bone-substitute [84]. Covering the perforated sinus membrane with PRF or collagen membrane in the rabbit sinus augmentation did not statistically affect the healing [85]. Placing a PRF membrane on top of the collagen membrane covering the lateral osteotomy site has been suggested to prevent the negative impact of the early PRF resorption [86]. Such a membrane combination might be worthwhile trying in periodontal defects.

The addition of L-PRF to other regenerative techniques has not been thoroughly explored. The basic concept for the application of APCs in periodontal defects is enhancement of the periodontal regeneration as adjuncts to standard regenerative techniques. In this context, there is insufficient evidence for the addition of L-PRF to EMD or GTR. The combined use of L-PRF and OGs is the mostly documented, though it should be tested more in contained and non-contained defects. With the combined PRF/OG, the graft helps in space maintenance, scaffolding and flap collapse prevention, which is important in non-contained defects. The increased defect fill found with the addition of PRF to the graft [63] might imply higher bone fill and therefore enhanced bone formation. In the combined PRF/OG, it has been suggested that PRF acts as a matrix allowing neo-angiogenesis, stem cell retention and migration of osteoprogenitor cells [84]. Several questions arise on the combined PRF/OG that only the histologic evaluation can answer, such as the nature of the tissues in the healed defect, the amount of newly formed bone, the amount of residual bone substitute, the contact between new bone and bone substitute and the possible acceleration of the bone formation process. Future studies should address the combined use of L-PRF, OG and GTR, as compared without L-PRF, since there is only one such study for PRF [87]. Such a comparison in non-contained defects would be challenging.

Furthermore, the papilla preservation techniques, single-flap approach, modified suturing techniques for complete flap closure and minimally invasive surgical approach should be studied in combination to PRF.

Finally, the role of autologous platelet concentrates as potential drug delivery systems for locally delivered biomolecules seems very promising and should be further explored.

The main limitations of the present study are as follows. Based on the available literature, meta-analysis was performed only for the adjunctive use of L-PRF to OFD and to OG in two- and/or three-wall endosseous defects. Concerning endosseous defects, meta-analysis was not feasible for the adjunctive use of L-PRF to GTR, EMD, and to small biomolecules. Concerning class II furcation defects, meta-analysis was not performed at all. Analysis for endosseous defects of unfavorable morphology, such as one- and/or two-wall defects, was not feasible. Analysis of the long-term adjunctive effect of L-PRF was not performed due to lack of relevant data.

## 5. Conclusions

Within the limitations of the present systematic review and meta-analysis of RCTs, conclusions are drawn as follows:

- For two- and/or three-wall endosseous defects and for class II furcation defects of systemically healthy non-smoking periodontitis patients, using L-PRF following OFD is a treatment option;
- The adjunctive use of L-PRF to OFD in two- and/or three-wall endosseous defects of systemically healthy non-smoking periodontitis patients is significantly beneficial for PPD reduction, CAL gain and DD reduction, as compared with OFD alone;
- The adjunctive use of L-PRF to OG in two- and/or three-wall endosseous defects of systemically healthy non-smoking chronic periodontitis patients is significantly beneficial for PPD reduction, CAL gain and DD reduction, as compared with OG alone.

Furthermore, the data showed the following:

- It seems that the addition of L-PRF to OFD in endosseous defects is more justified for the radiographic improvement expected to be achieved, than for the clinical one;
- For endosseous defects, the adjunctive use of L-PRF to GTR and EMD, as compared without L-PRF, has not been sufficiently documented;
- For endosseous defects, the adjunctive use of L-PRF to small biomolecules, such as metformin, has not been sufficiently documented;
- The adjunctive use of L-PRF to OFD in class II furcation defects of systemically healthy non-smoking periodontitis patients, seems to be significantly beneficial for PPD reduction, horizontal and vertical CAL gain and DD reduction, as compared with OFD alone;
- The adjunctive use of L-PRF to OG in class II furcation defects of systemically healthy non-smoking periodontitis patients, seems to be significantly beneficial for PPD reduction and CAL gain, as compared with OG alone;
- For furcation defects, the adjunctive use of L-PRF to GTR and EMD, as compared without L-PRF, has not been documented at all;
- For endosseous defects, further research is required on the adjunctive use of L-PRF to GTR and EMD, as compared without L-PRF;
- For furcation defects, further research is required on the adjunctive use of L-PRF to conventional regenerative techniques, as compared without L-PRF.

Concluding, the prevailing finding of the present systematic review and meta-analysis is that there is significant clinical and radiographic additive effectiveness of L-PRF to OFD and to OG in two- and/or three-wall periodontal endosseous defects of systemically healthy non-smokers, as compared without L-PRF. However, more studies must be conducted with longer periods of follow up and larger population, in order to achieve better statistical results.

## Figures and Tables

**Table 1:** Excluded studies.

Authors	Year	Reason of Exclusion
Lekovic et al. [23]	2012	No control group (Reason 2)
Pradeep et al. [24]	2012	Non-independence analysis unit (Reason 3)
Bajaj et al. [25]	2013	Mixed design clinical trial (Reason 1)
Elgendy et al. [26]	2015	Smokers included (Reason 4)
Shah et al. [27]	2015	Mixed design clinical trial (Reason 1)
Siddiqui et al. [28]	2016	Mixed design clinical trial (Reason 1)
Qiao et al. [29]	2016	Mixed design clinical trial (Reason 1)
Agarwal et al. [30]	2017	Mixed design clinical trial (Reason 1)
Bajaj et al. [31]	2017	Mixed design clinical trial (Reason 1)
Chatterjee et al. [32]	2017	Mixed design clinical trial (Reason 1)
Lohi et al. [33]	2017	Mixed design clinical trial (Reason 1)
Pradeep et al. [34]	2017	Mixed design clinical trial (Reason 1)
Betancourt et al. [35]	2017	Case report (Reason 5)
Cieplik et al. [36]	2018	Incomplete data (Reason 6)
Wanikar et al. [37]	2019	PRF was not used as an adjunct (Reason 7)
Pardo-Zamora et al. [38]	2019	Case series (Reason 5)
Lei et al. [39]	2019	Case report (Reason 5)
Gummaluri et al. [40]	2020	Mixed design clinical trial (Reason 1)

**Table 2:** Characteristics of included studies.

Endosseous Defects					
(L-PRF + OFD) vs. OFD Alone					
Author Year	Study Design Time	Population Characteristics	Interventions Groups	Parameters Evaluated	Outcomes (Test vs. Control Group)
**Sharma and Pradeep 2011 (b)** [41]	Parallel Time: 9 mo Ptis: Chronic 3 w	Smokers: Excluded Age: 35.34 ± 6.45 years Gender: 18 F/24 M Teeth treated: 69 *n* randomized (participants/teeth): 42/69 *n* evaluated (participants/teeth): 35/56	(L-PRF + OFD) vs. OFD Test: L-PRF + OFD (*n* = 28) Control: OFD (*n* = 28) L-PRF preparation: 3000 rpm × 10 min Clots, membrane	Clinical: PPD, CAL, GML Radiographic: DD	PPD reduction: (4.55 ± 1.87 mm) vs. (3.21 ± 1.64 mm) (*p* = 0.006) CAL gain: (3.331 ± 1.76 mm) vs. (2.77 ± 1.44 mm) (*p* = 0.2143) GML change: (−0.10 ± 0.08 mm) vs. (0.67 ± 0.46 mm) (*p* < 0.001) DD reduction: (2.50 ± 0.78 mm) vs. (0.09 ± 0.11 mm) (*p* < 0.001) DF: (48.26 ± 5.72%) vs. (1.80 ± 1.56%) (*p* < 0.001)
**Thorat et al., 2011** [42]	Parallel Time: 9 mo Ptis: Chronic 2–3 w	Smokers: Excluded Age: 31.12 ± 2.06 years Gender: 18 F/22 M Teeth treated: 40 *n* randomized (participants/teeth): 40/40 *n* evaluated (participants/teeth): 32/32	(L-PRF + OFD) vs. OFD Test: L-PRF + OFD (*n* = 16) Control: OFD (*n* = 16) L-PRF preparation: 400 g × 12 min Clots, membrane	Clinical: PPD, CAL, GML Radiographic: DD	PPD reduction: (4.69 ± 1.45 mm) vs. (3.56 ± 1.09 mm) (*p* < 0.05) CA L gain: (4.13 ± 1.63 mm) vs. (2.13 ± 1.71 mm) (*p* < 0.05) GML change: (−0.31 ± 0.95 mm) vs. (−1.31 ± 1.01 mm) (*p* < 0.05) DD reduction: (2.12 ± 0.69 mm) vs. (1.24 ± 0.69 mm) (*p* < 0.05)
**Ajwani et al., 2015** [43]	Split-mouth Time: 9 mo Ptis: NR 2–3 w	Smokers: Excluded Mean: 30.5 years Gender: 10 F/10 M Teeth treated: 40 *n* randomized (participants/teeth): 20/40 *n* evaluated (participants/teeth): 20/40	(L-PRF + OFD) vs. OFD Test: L-PRF + OFD (*n* = 20) Control: OFD (*n* = 20) L-PRF preparation: 3000 rpm × 10 min Clots, membrane	Clinical: PPD, RAL, GML Radiographic: CEJ-BOD, AC-BOD, CEJ-AC	PPD reduction: (1.90 ± 0.738 mm) vs. (1.60 ± 0.843 mm) (*p* = 0.408) RAL gain: (1.80 ± 0.632 mm) vs. (1.30 ± 0.675 mm) (*p* = 0.105) GML change: (−0.30 ± 0.483 mm) vs. (−0.30 ± 0.675 mm) (*p* = 0.08) CEJ-BOD change: (2.60 ± 1.101 mm) vs. (1.30 ± 0.422 mm) (*p* = 0.003) AC-BOD change: (1.45 ± 0.497 mm) vs. (0.80 ± 0.350 mm) (*p* = 0.003) CEJ-AC change: (1.20 ± 1.006 mm) vs. (0.50 ± 0.471 mm) (*p* = 0.062)
**Pradeep et al., 2015** [44]	Parallel Time: 9 mo Ptis: Chronic 3 w	Smokers: Excluded Age: 41 years Gender: 68 F/68 M Teeth treated: 64 (126 for all 4 groups) *n* randomized (participants/teeth): 64/64 (126/126 for all 4 groups) *n* evaluated (participants/teeth): 60/60 (120/120 for all 4 groups)	(L-PRF + OFD) vs. OFD Test 1: L-PRF + OFD (*n* = 30) Control: OFD (*n* = 30) L-PRF preparation: 3000 rpm × 10 min Clots, membrane Group 3 and 4 not included	Clinical: PPD, RAL, GML Radiographic: DD	PPD reduction: (4.00 ± 0.18 mm) vs. (3.00 ± 0.18) (*p* < 0.001) RAL gain: (4.03 ± 0.18 mm) vs. (2.96 ± 0.18 mm) (*p* < 0.001) GML change: (0.27 ± 0.007 mm) vs. (−0.06 ± 0.04 mm) (*p* < 0.001) DD reduction: (2.53 ± 0.30 mm) vs. (0.49 ± 0.27 mm) (*p* < 0.001) % DD reduction: (48.00 ± 0.0029%) vs. (9.14 ± 0.004%) (*p* < 0.001)
		Smokers: Excluded Age: 41 years Gender: 68 F/68 M Teeth treated: 62 (126 for all 4 groups) *n* randomized (participants/teeth): 62/62 (126/126 for all 4 groups) *n* evaluated (participants/teeth): 60/60 (120/120 for all 4 groups)	(L-PRF + 1% MF + OFD) vs. (1%MF + OFD) Test 2: L-PRF + 1% MF + OFD (*n* = 30) Control: 1%MF + OFD (*n* = 30) L-PRF preparation: 3000 rpm × 10 min Clots, membrane Group 1 and 2 not included	Clinical: PPD, RAL, GML Radiographic: DD	PPD reduction: (4.90 ± 0.30 mm) vs. (3.93 ± 0.25) (*p* = 0.084) RAL gain: (4.90 ± 0.30 mm) vs. (3.93 ± 0.25 mm) (*p* = 0.079) GML change: (0.33 ± 0.07 mm) vs. (0.27 ± 0.05 mm) (*p* = 0.420) DD reduction: (2.77 ± 0.30 mm) vs.(2.56 ± 0.28 mm) (*p* < 0.05) % DD reduction: (52.65 ± 0.031%) vs. (48.69 ± 0.026%) (*p* < 0.05)
**Kanoriya et al., 2016** [45]	Parallel Time: 9 mo Ptis: Chronic 3 w	Smokers: Excluded Age: 39 years Gender: 55 F/53 M Teeth treated: 64 (96 for all 3 groups) *n* randomized (participants/teeth): 64/64 (96/96 for all 3 groups) *n* evaluated (participants/teeth): 60/60 (90/90 for all 3 groups)	(L-PRF + OFD) vs. OFD Test group: L-PRF + OFD (*n* = 30) Control group: OFD alone (*n* = 30) L-PRF preparation: 3000 rpm × 10 min Clots, membrane Group 3 not included	Clinical: PPD, CAL, GML Radiographic: DD	PPD reduction: (3.70 ± 0.91 mm) vs. (2.86 ± 0.68 mm) (*p* < 0.05) CAL gain: (4.20 ± 0.66 mm) vs. (3.03 ± 0.18 mm) (*p* < 0.05) GML change: (0.24 ± 0.056 mm) vs. (−0.06 ± 0.07) (*p* < 0.05) DD reduction: (2.42 ± 0.21 mm) vs. (0.38 ± 0.26 mm) (*p* < 0.01) DF: (46.00 ± 1.89%) vs. (7.33 ± 4.86%) (*p* < 0.01)
**Chandradas et al., 2016** [46]	Parallel Time: 9 mo Ptis: Chronic 2–3 w	Smokers: Excluded Age range: 35–50 years Gender: 18 F/18 M Teeth treated: 24 (36 for all 3 groups) *n* randomized (participants/teeth): 24/24 (36/36 for all 3 groups) *n* evaluated (participants/teeth): 24/24 (36/36 for all 3 groups)	(L-PRF + OFD) vs. OFD Test: L-PRF + OFD (*n* = 12) Control: OFD alone (*n* = 12) L-PRF preparation: 3000 rpm × 12 min Membrane Group 2 not included	Clinical: PPD, RAL, GR Radiographic: DD	PPD reduction: (3.00 ± 1.21 mm) vs. (3.82 ± 0.75 mm) (*p* = 0.109) RAL gain: (3.27 ± 0.65 mm) vs. (2.25 ± 0.62 mm) (*p* = 0.003) GML change: (−0.18 ± 0.40 mm) vs. (−1.33 ± 0.78 mm) (*p* = 0.002) DD reduction: (2.30 ± 0.83 mm) vs. (1.22 ± 0.62 mm) (*p* = 0.001)
**Martande et al., 2016** [47]	Parallel Time: 9 mo Ptis: Chronic 3 w	Smokers: Excluded Mean age at baseline: 37.6 years Gender: 48 F/48 M Teeth treated: 64 (96 for all 3 groups) *n* randomized (participants/teeth): 64/64 (96/96 for all 3 groups) *n* evaluated (participants/teeth): 60/60 (90/90 for all 3 groups)	Comparison: L-PRF + OFD vs. OFD alone Test group: L-PRF + OFD (*n* = 30) Control group: OFD alone (*n* = 30) L-PRF preparation: 3000 rpm × 12–14 min Clots, membrane Group 3 not included	Clinical: PD, CAL, GML Radiographic: DD	PPD reduction: (3.76 ± 1.12 mm) vs. (2.76 ± 1.43 mm) (*p* = 0.01) CA gain L: (3.40 ± 1.13 mm) vs. (2.50 ± 1.33 mm) (*p* = 0.03) GML change: (0.22 ± 0.10 mm) vs. (0.06 ± 0.02 mm) (*p* < 0.001) DD reduction: (2.46 ± 0.33 mm) vs. (0.27 ± 0.19 mm) (*p* < 0.001) DF: (47.91 ± 4.79%) vs. (5.54 ± 1.71%) (*p* < 0.001)
**Pradeep et al., 2016** [48]	Parallel Time: 9 mo Ptis: Chronic 2–3 w	Smokers: Excluded Age: 35 years Gender: 45 F/45 M Teeth treated: 60 (90 for all 3 groups) *n* randomized (participants/teeth): 60/60 (90/90 for all 3 groups) *n* evaluated (participants/teeth): 60/60 (90/90 for all 3 groups)	(L-PRF + OFD) vs. OFD Test: L-PRF + OFD (*n* = 30) Control: OFD (*n* = 30) L-PRF preparation: 3000 rpm × 10 min Membrane	Clinical: PPD, CAL Radiographic: DD	PPD reduction: (4.03 ± 0.18 mm) vs. (3.10 ± 0.30 mm) (*p* < 0.001) CAL gain: (3.30 ± 0.65 mm) vs. (2.47 ± 0.77 mm) (*p* < 0.001) DD reduction: (3.17 ± 0.65 mm) vs. (1.43 ± 0.50 mm) (*p* < 0.001)
**Patel et al., 2017** [49]	Split-mouth Time:12 mo Ptis: Chronic 2–3 w	Smokers: Excluded Age: 44 ± 9 years Gender: 9 F/4 M Teeth treated: 26 *n* randomized (participants/teeth): 13/26 *n* evaluated (participants/teeth): 13/26	(L-PRF + OFD) vs. OFD Test: L-PRF + OFD (*n* = 13) Control: OFD (*n* = 13) L-PRF preparation: 3000 rpm × 10 min Membrane	Clinical: PPD, CAL Radiographic: DF	PPD reduction: (4.20 ± 1.69 mm) vs. (2.40 ± 0.84 mm) (*p* = 0.001) CAL gain: (3.70 ± 0.67 mm) vs. (2.10 ± 0.74 mm) (*p* = 0.001) DF: (45.18 ± 7.57%) vs. (21.6 ± 9.3%) (*p* = 0.001)
**(L-PRF + OG) vs. OG Alone**					
**Agarwal et al., 2016** [50]	Split-mouth Time:12 mo Ptis: Chronic 2–3 w	Smokers: Excluded Age: 52 ± 7 years Gender: 14 F/18 M Teeth treated: 64 *n* randomized (participants/teeth): 32/64 *n* evaluated (participants/teeth): 30/60	(L-PRF + DFDBA) vs. (DFDBA + saline) Test: (L-PRF + DFDBA) (*n* = 30) Control: (DFDBA + saline) (*n* = 30) L-PRF preparation: 400 g × 12 min Clots, membrane	Clinical: PPD, CAL, REC Radiographic: CEJ-AC, AC-BOD, CEJ-BOD	PPD reduction: (4.15 ± 0.84 mm) vs. (3.60 ± 0.15 mm) (*p* < 0.05) CAL gain: (3.73 ± 0.74 mm) vs. (2.61 ± 0.68 mm) (*p* < 0.001) CEJ-AC change: (−0.23 ± 0.25 mm) vs. (−0.26 ± 0.25 mm) (*p* = 0.613) AC-BOD change: (3.73 ± 0.63 mm) vs. (2.75 ± 0.57) (*p* < 0.001) CEJ-BOD change: (3.50 ± 0.67 mm) vs. (2.49 ± 0.64 mm) (*p* < 0.001)
**Naqvi et al., 2017** [51]	Split-mouth Time: 9 mo Ptis: Chronic 2–3 w	Smokers: Excluded Age: 20–50 years Gender: 3 F/7 M Teeth treated: 20 *n* randomized (participants/teeth): 10/20 *n* evaluated (participants/teeth): 10/20	(L-PRF + BGP) vs. BGP Test: (L-PRF + BGP) (*n* = 10) Control: BGP (*n* = 10) L-PRF preparation: 3000 rpm × 10 min Membrane	Clinical: PPD, CAL Radiographic: DD	PPD reduction: (3.20 ± 2.30 mm) vs. (3.15 ± 1.06 mm) (*p* = 0.117) CAL gain: (4.10 ± 1.73 mm) vs. (3.15 ± 1.06 mm) (*p* = 0.155) DD reduction: (7.10 ± 1.37 mm) vs. (5.70 ± 1.64 mm) (*p* = 0.043)
**Sezgin et al., 2017** [52]	Split-mouth Time: 6 mo Ptis: Chronic 2–3 w	Smokers: Excluded Age: 38–61 years Gender: 7 F/8 M Teeth treated: 30 *n* randomized (participants/teeth): 21/42 *n* evaluated (participants/teeth): 5/30	(L-PRF + ABBM) vs. ABBM Test: (L-PRF + ABBM) (*n* = 15) Control: ABBM (*n* = 15) L-PRF preparation: 2700 rpm × 12 min Clots, membrane	Clinical: PPD, CAL, GR Radiographic: DD, vertical bone loss, defect angle	PPD reduction: (4.93 ± 1.22 mm) vs. (4.21 ± 1.21 mm) (*p* > 0.05) CAL gain: (4.47 ± 1.60 mm) vs. (3.27 ± 1.34 mm) (*p* < 0.05) GR: (0.46 ± 0.83 mm) vs. (0.94 ± 0.70 mm) (*p* > 0.05) DD reduction: (2.55 ± 1.15 mm) vs. (1.98 ± 0.80 mm) (*p* > 0.05)
**Bodhare et al., 2019** [53]	Split-mouth Time: 6 mo Ptis: Chronic 2–3 w	Smokers: Excluded Age: 35.9 years Gender: 9 F/11 M Teeth treated: 40 *n* randomized (participants/teeth): 20/40 *n* evaluated (participants/teeth): 20/40	(L-PRF + BG + OFD) vs. (BG + OFD) Test: (L-PRF + BG + OFD) (*n* = 20) Control: (BG + OFD) (*n* = 20) L-PRF preparation: 3000 rpm × 10 min Clots, membrane	Clinical: PPD, CAL, GML Radiographic: CEJ-BOD, CEJ-AC, AC-BOD, defect width (mesio-distal, bucco-lingual)	PPD reduction: (5.75 ± 1.16 mm) vs. (5.65 ± 1.66 mm) (*p* = 0.82) CAL gain: (5.05 ± 1.09 mm) vs. (4.20 ± 1.70 mm) (*p* = 0.02) GML change: (−0.80 ± 0.61 mm) vs. (−1.95 ± 1.09 mm) (*p* < 0.001) CEJ-BOD change: (3.30 ± 1.10 mm) vs. (2.49 ± 0.99 mm) (*p* = 0.02) AC-BOD change: (3.51 ± 1.71 mm) vs. (2.56 ± 0.94 mm) (*p* = 0.0077) CEJ-AC change: (0.13 ± 0.22 mm) vs. (0.33 ± 0.37 mm) (*p* = 0.2705) Defect width: Mesio-distal: (0.70 ± 0.68 mm) vs. (0.45 ± 0.18 mm) (*p* = 0.0047) Bucco-lingual: (1.60 ± 0.27 mm) vs. (1.33 ± 0.44 mm) (*p* = 0.00319)
**(L-PRF + GTR) vs. GTR Alone**					
**Panda et al., 2016** [54]	Split-mouth Time: 9 mo Ptis: Chronic 3 w	Smokers: Excluded Age: 38.12 ± 2.06 years Gender: 8 F/10 M Teeth treated: 36 *n* randomized (participants/teeth): 18/36 *n* evaluated (participants/teeth): 6/32	(L-PRF + GTR) vs. GTR Test: (L-PRF + GTR) (*n* = 16) Control: GTR (*n* = 16) L-PRF preparation: 3000 rpm × 10 min	Clinical: PPD, CAL, GML Radiographic: DD	PPD: (3.88 ± 1.15 mm) vs. (3.19 ± 1.33 mm) (*p* = 0.13) CAL gain: (4.44 ± 1.50 mm) vs. (3.38 ± 1.45 mm) (*p* = 0.05) GML change: (1.00 ± 0.67 mm) vs. (0.29 ± 0.49 mm) (*p* = 0.03) DD reduction: (2.10 ± 0.64 mm) vs. (0.80 ± 0.28 mm) (*p* < 0.001)
**(L-PRF + EMD) vs. EMD Alone**					
**Aydemir et al., 2016** [55]	Split-mouth Time: 6 mo Ptis: Chronic 1-,2-,3-w	Smokers: Excluded Age: 38.53 ± 9.24 years Gender: 14 F/14 M Teeth treated: 56 *n* randomized (participants/teeth): 28/56 *n* evaluated (participants/teeth): 25/49	(L-PRF + EMD) vs. EMD Test: (L-PRF + EMD) Control: EMD L-PRF preparation: 400 g × 10 min Membrane	Clinical: PPD, CAL. GR Radiographic: DD, CEJ-BOD, defect width, defect angle	PPD reduction: (4.00 ± 1.38 mm) vs. (3.88 ± 1.26 mm) (*p* = 0.746) CAL gain: (3.42 ± 1.28 mm) vs. (3.29 ± 1.30 mm) (*p* = 0.718) GR: (0.71 ± 0.86 mm) vs. (0.58 ± 0.78 mm) (*p* = 0.574) DD reduction: (1.17 ± 0.86 mm) vs. (1.19 ± 1.25 mm) (*p* = 0.937)
**Furcation Defects**					
**(L-PRF + OFD) vs. OFD Alone**					
**Sharma and Pradeep 2011 (a)** [56]	Split-mouth Time: 9 mo Ptis: NR Class II	Smokers: excluded Age: 34.2 years Gender: 8 F/10 M Teeth treated: 36 *n* randomized (participants/teeth): 8/36 *n* evaluated (participants/teeth): 8/36	(OFD + L-PRF) vs. OFD Test: (OFD + L-PRF) (*n* = 36) Control: OFD (*n* = 36) L-PRF preparation: 3000 rpm × 10 min Clots, membrane	Clinical: PPD, RVCAL, RHCAL, GML Radiographic: DD, DF	PPD reduction: (4.056 ± 0.416 mm) vs. (2.889 ± 0.676 mm) (*p* < 0.001) RVCAL gain: (2.333 ± 0.485 mm) vs. (1.278 ± 0.461 mm) (*p* < 0.001) RHCAL gain: (2.667 ± 0.594 mm) vs. (1.889 ± 0.758) (*p* = 0.002) GM Lchange: (0.344 ± 0.086 mm) vs. (0.756 ± 0.115 mm) (*p* < 0.001) DD reduction: (2.006 ± 0.163 mm) vs. (0.622 ± 0.216) (*p* < 0.001) DF: (50.8 ± 6.24%) vs. (16.7 ± 6.42%) (*p* < 0.001)
**Kanoriya et al., 2017** [57]	Parallel Time: 9 mo Ptis: Chronic Class II	Smokers: Excluded Age: 39.45 ± 5.20 years (control), 38.30 ± 5.35 years (test) Gender: 36 F/36 M Teeth treated: 52 (78 for all 3 groups) *n* randomized (participants/teeth): 52/52 (78/78 for all 3 groups) *n* evaluated (participants/teeth): 47/47 (72/72 for all 3 groups)	(L-PRF + OFD) vs. OFD Test: (L-PRF + OFD) (*n* = 23) Control: OFD (*n* = 24) L-PRF preparation: 3000 rpm × 10 min Clots, membrane Group 3 not included	Clinical: PPD, RVAL, RVHL Radiographic: DD	PPD reduction: (3.69 ± 0.76 mm) vs. (2.41 ± 0.77 mm) (*p* < 0.001) RVAL gain: (3.39 ± 0.49 mm) vs. (2.33 ± 0.48 mm) (*p* < 0.001) RVHL gain: (2.86 ± 0.062 mm) vs. (2.04 ± 0.35 mm) (*p* < 0.001) DD reduction: (2.59 ± 0.32 mm) vs. (0.52 ± 0.19 mm) (*p* < 0.001)
**(L-PRF + OG) vs. OG Alone**					
**Basireddy et al., 2019** [58]	Split-mouth Time: 9 mo Ptis: Chronic Class II	Smokers: Excluded Age range: 30–50 years Gender: NR Teeth treated: 110 *n* randomized (participants/teeth): 14/28 *n* evaluated (participants/teeth): 14/28	(L-PRF + DFDBA + OFD) vs. (DFDBA + OFD) Test: (L-PRF + DFDBA + OFD) (*n* = 14) Control: (DFDBA + OFD) (*n* = 14) L-PRF preparation: 3000 rpm × 10 min Membrane	Clinical: PD, RVCAL, RHCAL, GML Radiographic (CBCT): VDD, HDD	PD reduction: (2.50 ± 0.519 mm) vs. (2.336 ± 0.497 mm) (*p* > 0.05) RVCAL gain: (2.36 ± 0.497 mm) vs. (1.79 ± 0.802 mm) (*p* > 0.05) RHCAL gain: (4.57 ± 1.697 mm) vs. (1.50 ± 1.092 mm) (*p* < 0.001) GML change: (−0.21 ± 0.426 mm) vs. (−0.79 ± 0.579 mm) (*p* < 0.05) VDD reduction: (46.36 ± 22.7%) vs. (42.36 ± 21.35%) (*p* > 0.05) HDD reduction: (38.20 ± 12.57%) vs. (37.99 ± 13.56%) (*p* > 0.05)
**Dambhare et al., 2019** [59]	Parallel Time:12 mo Ptis: Chronic Class II	Smokers: Excluded Age: 40 ± 4.29 years Gender: NR Teeth treated: 24 *n* randomized (participants/teeth): 24/24 *n* evaluated (participants/teeth): 24/24	(L-PRF + HA + β-TCP + OFD) vs. (HA + β-TCP + OFD) Test: (L-PRF + HA + β-TCP + OFD) (*n* = 12) Control: (HA + β-TCP + OFD) (*n* = 12) L-PRF preparation: 400 g × 12 min Clots, membrane	Clinical: PPD, CAL, REC	PPD reduction: (2.00 ± 0.73 mm) vs. (0.50 ± 0.52 mm) (*p* < 0.05) CAL gain: (3.33 ± 0.83 mm) vs. (2.00 ± 0.85 mm) (*p* < 0.05) REC: (1.0 ± 1.12 mm) vs. (1.34 ± 1.07 mm) (*p* > 0.05)

Abbreviations: *n*: number; mo: months; y: year; Ptis: periodontitis type; w: number of osseous walls; Class II: class II furcation defect; OFD: open flap debridement; L-PRP: leucocyte-and platelet-rich plasma; PRP: platelet-rich plasma; min: minutes; rpm: revolution per minute; PPD: probing pocket depth; CAL: clinical attachment level; GML: gingival margin level; DD: radiographic defect depth; %DF: % radiographic defect fill; RAL: relative attachment gain; CEJ-BOD: cementoenamel junction-base of the defect; AC-BOD: alveolar crest-base of the defect; CEJ-AC: cementoenamel junction-alveolar crest; REC (or GR): gingival recession; RVCAL: relevant vertical clinical attachment level; RHCAL: relevant horizontal clinical attachment level; VDD: vertical defect depth; HDD: horizontal defect depth; VDD: % vertical radiographic defect depth reduction; HDD: % horizontal radiographic defect depth reduction; OG: osseous graft; DFDBA: demineralized freeze-dried bone allograft; ABBM: anorganic bovine bone mineral; BGP: bioactive glass putty; BG: bioactive bone alloplast; HA: hydroxyapatite; β-TCP: beta-tricalcium phosphate; GTR: guided tissue regeneration; EMD: enamel matrix derivative; NR: non-reported; 400 g: 400 g centrifugation force (corresponding to 2700 rpm [13]); Clots, membrane: L-PRF used in clot and in membrane form; Membrane: L-PRF used in membrane form; vs.: versus.

## Data Availability

The data presented in this study are available in Supplementary Materials.

## Supplementary Materials

The following supporting information can be downloaded at: https://www.mdpi.com/article/10.3390/ma15062088/s1, Listings: Search Algorithms, Figure S1: Funnel plot of comparison: 1 Endosseous Defects, outcome: 1.1. Probing Pocket Depth Reduction, Figure S2: Forest plot of comparison: 2 Endosseous Defects, outcome: 2.1. Clinical Attachment Level Gain, Figure S3: Forest plot of comparison: 3 Endosseous Defects, outcome: 3.1. Defect Depth Reduction, Figure S4: Forest plot of comparison: 4 Furcation Defects, outcome: 4.1. Probing Pocket Depth Reduction, Figure S5: Forest plot of comparison: 5 Furcation Defects, outcome: 5.1. Clinical Attachment Level Gain, Figure S6: Forest plot of comparison: 6 Furcation Defects, outcome: 6.1. Defect Depth Reduction.

## References

[1] Wang H.-L., Greenwell H., Fiorellini J., Giannobile W., Offenbacher S., Salkin L., Townsend C., Sheridan P., Genco R.J., Research S. (2005). Periodontal Regeneration. J. Periodontol..

[2] Sculean A., Nikolidakis D., Nikou G., Ivanovic A., Chapple I.L.C., Stavropoulos A. (2015). Biomaterials for Promoting Periodontal Regeneration in Human Intrabony Defects: A Systematic Review. Periodontology.

[3] Nibali L., Koidou V.P., Nieri M., Barbato L., Pagliaro U., Cairo F. (2020). Regenerative Surgery versus Access Flap for the Treatment of Intra-Bony Periodontal Defects: A Systematic Review and Meta-Analysis. J. Clin. Periodontol..

[4] Onicas M.I., Narita L.E., Mester A., Onisor F., Mancini L. (2021). Platelet-Rich Fibrin: A Viable Therapy for Endodontic-Periodontal Lesions? A Preliminary Assessment. Appl. Sci..

[5] Dohan Ehrenfest D.M., Rasmusson L., Albrektsson T. (2009). Classification of Platelet Concentrates: From Pure Platelet-Rich Plasma (P-PRP) to Leucocyte- and Platelet-Rich Fibrin (L-PRF). Trends Biotechnol..

[6] Ghanaati S., Booms P., Orlowska A., Kubesch A., Lorenz J., Rutkowski J., Les C., Sader R., Kirkpatrick C.J., Choukroun J. (2014). Advanced Platelet-Rich Fibrin: A New Concept for Cell-Based Tissue Engineering by Means of Inflammatory Cells. J. Oral Implantol..

[7] Tunali M., Özdemir H., Küçükodaci Z., Akman S., Yaprak E., Toker H., Firatli E. (2014). A Novel Platelet Concentrate: Titanium-Prepared Platelet-Rich Fibrin. BioMed Res. Int..

[8] Miron R.J., Fujioka-Kobayashi M., Hernandez M., Kandalam U., Zhang Y., Ghanaati S., Choukroun J. (2017). Injectable Platelet Rich Fibrin (i-PRF): Opportunities in Regenerative Dentistry?. Clin. Oral Investig..

[9] Miron R.J., Chai J., Zhang P., Li Y., Wang Y., de Almeida Barros Mourão C.F., Sculean A., Fujioka Kobayashi M., Zhang Y. (2020). A Novel Method for Harvesting Concentrated Platelet-Rich Fibrin (C-PRF) with a 10-Fold Increase in Platelet and Leukocyte Yields. Clin. Oral Investig..

[10] Choukroun J., Abba F., Schoeffler C., Vervelle A. (2001). Une Opportunite En Paro-Implantologie: Le PRF. Implantodontie.

[11] Dohan D.M., Choukroun J., Diss A., Dohan S.L., Dohan A.J.J., Mouhyi J., Gogly B. (2006). Platelet-Rich Fibrin (PRF): A Second-Generation Platelet Concentrate. Part III: Leucocyte Activation: A New Feature for Platelet Concentrates?. Oral Surg. Oral Med. Oral Pathol. Oral Radiol. Endodontol..

[12] Dohan D.M., Choukroun J., Diss A., Dohan S.L., Dohan A.J.J., Mouhyi J., Gogly B. (2006). Platelet-Rich Fibrin (PRF): A Second-Generation Platelet Concentrate. Part II: Platelet-Related Biologic Features. Oral Surg. Oral Med. Oral Pathol. Oral Radiol. Endodontol..

[13] Khorshidi H., Raoofi S., Bagheri R., Banihashemi H. (2016). Comparison of the Mechanical Properties of Early Leukocyte- and Platelet-Rich Fibrin versus PRGF/Endoret Membranes. Int. J. Dent..

[14] Dohan Ehrenfest D.M., Pinto N.R., Pereda A., Jiménez P., del Corso M., Kang B.S., Nally M., Lanata N., Wang H.L., Quirynen M. (2018). The Impact of the Centrifuge Characteristics and Centrifugation Protocols on the Cells, Growth Factors, and Fibrin Architecture of a Leukocyte- and Platelet-Rich Fibrin (L-PRF) Clot and Membrane. Platelets.

[15] Kobayashi E., Flückiger L., Fujioka-Kobayashi M., Sawada K., Sculean A., Schaller B., Miron R.J. (2016). Comparative Release of Growth Factors from PRP, PRF, and Advanced-PRF. Clin. Oral Investig..

[16] Dohan Ehrenfest D.M., de Peppo G.M., Doglioli P., Sammartino G. (2009). Slow Release of Growth Factors and Thrombospondin-1 in Choukroun’s Platelet-Rich Fibrin (PRF): A Gold Standard to Achieve for All Surgical Platelet Concentrates Technologies. Growth Factors.

[17] Bhattacharya H., Gummaluri S., Astekar M., Gummaluri R. (2020). Novel Method of Determining the Periodontal Regenerative Capacity of T-PRF and L-PRF: An Immunohistochemical Study. Dent. Med. Probl..

[18] Zhang J., Yin C., Zhao Q., Zhao Z., Wang J., Miron R.J., Zhang Y. (2020). Anti-inflammation Effects of Injectable Platelet-rich Fibrin via Macrophages and Dendritic Cells. J. Biomed. Mater. Res. Part A.

[19] Wang Z., Mudalal M., Sun Y., Liu Y., Wang J., Wang Y., Sun X., Zhou Y. (2020). The Effects of Leukocyte-Platelet Rich Fibrin (L-PRF) on Suppression of the Expressions of the Pro-Inflammatory Cytokines, and Proliferation of Schwann Cell, and Neurotrophic Factors. Sci. Rep..

[20] Fujioka-Kobayashi M., Katagiri H., Kono M., Schaller B., Zhang Y., Sculean A., Miron R.J. (2020). Improved Growth Factor Delivery and Cellular Activity Using Concentrated Platelet-Rich Fibrin (C-PRF) When Compared with Traditional Injectable (i-PRF) Protocols. Clin. Oral Investig..

[21] Higgins J.P.T., Thomas J., Chandler J., Cumpston M., Li T., Page M.J., Welch V.A. (2019). Starting a review. Cochrane Handbook for Systematic Reviews of Interventions.

[22] Higgins J.P.T., Thomas J., Chandler J., Cumpston M., Li T., Page M.J., Welch V.A. (2019). Considering bias and conflicts of interest among the included studies. Cochrane Handbook for Systematic Reviews of Interventions.

[23] Lekovic V., Milinkovic I., Aleksic Z., Jankovic S., Stankovic P., Kenney E.B., Camargo P.M. (2012). Platelet-Rich Fibrin and Bovine Porous Bone Mineral vs. Platelet-Rich Fibrin in the Treatment of Intrabony Periodontal Defects. J. Periodontal Res..

[24] Pradeep A.R., Rao N.S., Agarwal E., Bajaj P., Kumari M., Naik S.B. (2012). Comparative Evaluation of Autologous Platelet-Rich Fibrin and Platelet-Rich Plasma in the Treatment of 3-Wall Intrabony Defects in Chronic Periodontitis: A Randomized Controlled Clinical Trial. J. Periodontol..

[25] Bajaj P., Pradeep A.R., Agarwal E., Rao N.S., Naik S.B., Priyanka N., Kalra N. (2013). Comparative Evaluation of Autologous Platelet-Rich Fibrin and Platelet-Rich Plasma in the Treatment of Mandibular Degree II Furcation Defects: A Randomized Controlled Clinical Trial. J. Periodontal Res..

[26] Elgendy E.A., Shady T.E.A. (2015). Clinical and Radiographic Evaluation of Nanocrystalline Hydroxyapatite with or without Platelet-Rich Fibrin Membrane in the Treatment of Periodontal Intrabony Defects. J. Indian Soc. Periodontol..

[27] Shah M., Patel J., Dave D., Shah S. (2015). Comparative Evaluation of Platelet-Rich Fibrin with Demineralized Freeze-Dried Bone Allograft in Periodontal Infrabony Defects: A Randomized Controlled Clinical Study. J. Indian Soc. Periodontol..

[28] Siddiqui Z.R., Jhingran R., Bains V.K., Srivastava R., Madan R., Rizvi I. (2016). Comparative Evaluation of Platelet-Rich Fibrin versus Beta-Tri-Calcium Phosphate in the Treatment of Grade II Mandibular Furcation Defects Using Cone-Beam Computed Tomography. Eur. J. Dent..

[29] Qiao J., Duan J., Zhang Y., Chu Y., Sun C. (2016). The Effect of Concentrated Growth Factors in the Treatment of Periodontal Intrabony Defects. Future Sci. OA.

[30] Agrawal I., Chandran S., Nadig P. (2017). Comparative Evaluation of the Efficacy of Platelet-Rich Fibrin and Calcium Phosphosilicate Putty Alone and in Combination in the Treatment of Intrabony Defects: A Randomized Clinical and Radiographic Study. Contemp. Clin. Dent..

[31] Bajaj P., Agarwal E., Rao N.S., Naik S.B., Pradeep A.R., Kalra N., Priyanka N., Kumari M. (2017). Autologous Platelet-Rich Fibrin in the Treatment of 3-Wall Intrabony Defects in Aggressive Periodontitis: A Randomized Controlled Clinical Trial. J. Periodontol..

[32] Chatterjee A., Pradeep A.R., Garg V., Yajamanya S., Ali M.M., Priya V.S. (2017). Treatment of Periodontal Intrabony Defects Using Autologous Platelet-Rich Fibrin and Titanium Platelet-Rich Fibrin: A Randomized, Clinical, Comparative Study. J. Investig. Clin. Dent..

[33] Lohi H.S., Nayak D.G., Uppoor A.S. (2017). Comparative Evaluation of the Efficacy of Bioactive Ceramic Composite Granules Alone and in Combination with Platelet Rich Fibrin in the Treatment of Mandibular Class II Furcation Defects: A Clinical and Radiographic Study. J. Clin. Diagn. Res. JCDR.

[34] Pradeep A.R., Bajaj P., Rao N.S., Agarwal E., Naik S.B. (2017). Platelet-Rich Fibrin Combined with a Porous Hydroxyapatite Graft for the Treatment of 3-Wall Intrabony Defects in Chronic Periodontitis: A Randomized Controlled Clinical Trial. J. Periodontol..

[35] Betancourt P., Elgueta R., Fuentes R. (2017). Treatment of Endo-Periodontal Lesion Using Leukocyte-Platelet-Rich Fibrin. A Case Report. Colomb. Med. (Cali Colomb.).

[36] Cieplik F., Tabenski L., Hiller K.-A., Schmalz G., Buchalla W., Christgau M. (2018). Influence of Autogenous Platelet Concentrate on Combined GTR/Graft Therapy in Intra-Bony Defects: A 13-Year Follow-up of a Randomized Controlled Clinical Split-Mouth Study. J. Clin. Periodontol..

[37] Wanikar I., Rathod S., Kolte A.P. (2019). Clinico-Radiographic Evaluation of 1% Alendronate Gel as an Adjunct and Smart Blood Derivative Platelet Rich Fibrin in Grade II Furcation Defects. J. Periodontol..

[38] Pardo-Zamora G., Moreno-Rodríguez J.A., Ortiz-Ruíz A.J. (2021). Non-Incised Papilla Surgical Approach and Leukocyte Platelet-Rich Fibrin in Periodontal Reconstruction of Deep Intrabony Defects: A Case Series. Int. J. Environ. Res. Public Health.

[39] Lei L., Yu Y., Ke T., Sun W., Chen L. (2019). The Application of Three-Dimensional Printing Model and Platelet-Rich Fibrin Technology in Guided Tissue Regeneration Surgery for Severe Bone Defects. J. Oral Implantol..

[40] Gummaluri S.S., Bhattacharya H.S., Astekar M., Cheruvu S. (2020). Evaluation of Titanium-Prepared Platelet-Rich Fibrin and Leucocyte Platelet-Rich Fibrin in the Treatment of Intra-Bony Defects: A Randomized Clinical Trial. J. Dent. Res. Dent. Clin. Dent. Prospect..

[41] Sharma A., Pradeep A.R. (2011). Treatment of 3-Wall Intrabony Defects in Patients with Chronic Periodontitis with Autologous Platelet-Rich Fibrin: A Randomized Controlled Clinical Trial. J. Periodontol..

[42] Thorat M., Pradeep A.R., Pallavi B. (2011). Clinical Effect of Autologous Platelet-Rich Fibrin in the Treatment of Intra-Bony Defects: A Controlled Clinical Trial. J. Clin. Periodontol..

[43] Ajwani H., Shetty S., Gopalakrishnan D., Kathariya R., Kulloli A., Dolas R.S., Pradeep A.R. (2015). Comparative Evaluation of Platelet-Rich Fibrin Biomaterial and Open Flap Debridement in the Treatment of Two and Three Wall Intrabony Defects. J. Int. Oral Health JIOH.

[44] Pradeep A.R., Nagpal K., Karvekar S., Patnaik K., Naik S.B., Guruprasad C.N. (2015). Platelet-Rich Fibrin with 1% Metformin for the Treatment of Intrabony Defects in Chronic Periodontitis: A Randomized Controlled Clinical Trial. J. Periodontol..

[45] Kanoriya D., Pradeep A.R., Singhal S., Garg V., Guruprasad C.N. (2016). Synergistic Approach Using Platelet-Rich Fibrin and 1% Alendronate for Intrabony Defect Treatment in Chronic Periodontitis: A Randomized Clinical Trial. J. Periodontol..

[46] Chandradas N., Ravindra S., Rangaraju V., Jain S., Dasappa S. (2016). Efficacy of Platelet Rich Fibrin in the Treatment of Human Intrabony Defects with or without Bone Graft: A Randomized Controlled Trial. J. Int. Soc. Prev. Community Dent..

[47] Martande S.S., Kumari M., Pradeep A.R., Singh S.P., Suke D.K., Guruprasad C.N. (2016). Platelet-Rich Fibrin Combined with 1.2% Atorvastatin for Treatment of Intrabony Defects in Chronic Periodontitis: A Randomized Controlled Clinical Trial. J. Periodontol..

[48] Pradeep A.R., Garg V., Kanoriya D., Singhal S. (2016). Platelet-Rich Fibrin with 1.2% Rosuvastatin for Treatment of Intrabony Defects in Chronic Periodontitis: A Randomized Controlled Clinical Trial. J. Periodontol..

[49] Patel G.K., Gaekwad S.S., Gujjari S.K., Veerendra Kumar S.C. (2017). Platelet-Rich Fibrin in Regeneration of Intrabony Defects: A Randomized Controlled Trial. J. Periodontol..

[50] Agarwal A., Gupta N.D., Jain A. (2016). Platelet Rich Fibrin Combined with Decalcified Freeze-Dried Bone Allograft for the Treatment of Human Intrabony Periodontal Defects: A Randomized Split Mouth Clinical Trail. Acta Odontol. Scand..

[51] Naqvi A., Gopalakrishnan D., Bhasin M.T., Sharma N., Haider K., Martande S. (2017). Comparative Evaluation of Bioactive Glass Putty and Platelet Rich Fibrin in the Treatment of Human Periodontal Intrabony Defects: A Randomized Control Trial. J. Clin. Diagn. Res..

[52] Sezgin Y., Uraz A., Taner I.L., Çulhaoğlu R. (2017). Effects of Platelet-Rich Fibrin on Healing of Intra-Bony Defects Treated with Anorganic Bovine Bone Mineral. Braz. Oral Res..

[53] Bodhare G.H., Kolte A.P., Kolte R.A., Shirke P.Y. (2019). Clinical and Radiographic Evaluation and Comparison of Bioactive Bone Alloplast Morsels When Used Alone and in Combination with Platelet-rich Fibrin in the Treatment of Periodontal Intrabony Defects—A Randomized Controlled Trial. J. Periodontol..

[54] Panda S., Sankari M., Satpathy A., Jayakumar D., Mozzati M., Mortellaro C., Gallesio G., Taschieri S., del Fabbro M. (2016). Adjunctive Effect of Autologus Platelet-Rich Fibrin to Barrier Membrane in the Treatment of Periodontal Intrabony Defects. J. Craniofacial Surg..

[55] Aydemir Turkal H., Demirer S., Dolgun A., Keceli H.G. (2016). Evaluation of the Adjunctive Effect of Platelet-Rich Fibrin to Enamel Matrix Derivative in the Treatment of Intrabony Defects. Six-Month Results of a Randomized, Split-Mouth, Controlled Clinical Study. J. Clin. Periodontol..

[56] Sharma A., Pradeep A.R. (2011). Autologous Platelet-Rich Fibrin in the Treatment of Mandibular Degree II Furcation Defects: A Randomized Clinical Trial. J. Periodontol..

[57] Kanoriya D., Pradeep A.R., Garg V., Singhal S. (2017). Mandibular Degree II Furcation Defects Treatment with Platelet-Rich Fibrin and 1% Alendronate Gel Combination: A Randomized Controlled Clinical Trial. J. Periodontol..

[58] Basireddy A., Prathypaty S.K., Yendluri D.B., Potharaju S.P. (2019). Demineralized Freeze-Dried Bone Allograft with or without Platelet-Rich Fibrin in the Treatment of Mandibular Degree II Furcation Defects: A Clinical and Cone Beam Computed Tomography Study. J. Indian Soc. Periodontol..

[59] Dambhare A., Bhongade M.L., Dhadse P.V., Sehdev B., Ganji K.K., Thakare K., Hiroshi M., Sugita Y., Maeda H., Alam M.K. (2019). A Randomized Controlled Clinical Study of Autologous Platelet Rich Fibrin (PRF) in Combination with HA and Beta-TCP or HA and Beta-TCP Alone for Treatment of Furcation Defects. J. Hard Tissue Biol..

[60] Del Fabbro M., Panda S., Jayakumar N.D., Sankari M., Varghese S., Ramamoorthi S., Ceci C., Ceresoli V., Taschieri S. (2014). Autologous Platelet Concentrates for Treatment of Periodontal Defects. Cochrane Database Syst. Rev..

[61] Castro A.B., Meschi N., Temmerman A., Pinto N., Lambrechts P., Teughels W., Quirynen M. (2017). Regenerative Potential of Leucocyte- and Platelet-Rich Fibrin. Part A: Intra-Bony Defects, Furcation Defects and Periodontal Plastic Surgery. A Systematic Review and Meta-Analysis. J. Clin. Periodontol..

[62] Li A., Yang H., Zhang J., Chen S., Wang H., Gao Y. (2019). Additive Effectiveness of Autologous Platelet-Rich Fibrin in the Treatment of Intrabony Defects. Medicine.

[63] Miron R.J., Moraschini V., Fujioka-Kobayashi M., Zhang Y., Kawase T., Cosgarea R., Jepsen S., Bishara M., Canullo L., Shirakata Y. (2021). Use of Platelet-Rich Fibrin for the Treatment of Periodontal Intrabony Defects: A Systematic Review and Meta-Analysis. Clin. Oral Investig..

[64] Chen L., Ding Y., Cheng G., Meng S. (2021). Use of Platelet-Rich Fibrin in the Treatment of Periodontal Intrabony Defects: A Systematic Review and Meta-Analysis. BioMed Res. Int..

[65] Castro A.B., Meschi N., Temmerman A., Pinto N., Lambrechts P., Teughels W., Quirynen M. (2017). Regenerative Potential of Leucocyte- and Platelet-Rich Fibrin. Part B: Sinus Floor Elevation, Alveolar Ridge Preservation and Implant Therapy. A Systematic Review. J. Clin. Periodontol..

[66] Panda S., Karanxha L., Goker F., Satpathy A., Taschieri S., Francetti L., Das A.C., Kumar M., Panda S., Del Fabbro M. (2019). Autologous Platelet Concentrates in Treatment of Furcation Defects—A Systematic Review and Meta-Analysis. Int. J. Mol. Sci..

[67] Tarallo F., Mancini L., Pitzurra L., Bizzarro S., Tepedino M., Marchetti E. (2020). Use of Platelet-Rich Fibrin in the Treatment of Grade 2 Furcation Defects: Systematic Review and Meta-Analysis. J. Clin. Med..

[68] Ustaoğlu G., Aydin Z.U., Özelçi F. (2020). Comparison of GTR, T-PRF and Open-Flap Debridement in the Treatment of Intrabony Defects with Endo-Perio Lesions: A Randomized Controlled Trial. Med. Oral. Patol. Oral. Cir. Buca.

[69] Smaïl-Faugeron V., Fron-Chabouis H., Courson F., Durieux P. (2014). Comparison of Intervention Effects in Split-Mouth and Parallel-Arm Randomized Controlled Trials: A Meta-Epidemiological Study. BMC Med. Res. Methodol..

[70] Lesaffre E., Philstrom B., Needleman I., Worthington H. (2009). The Design and Analysis of Split-Mouth Studies: What Statisticians and Clinicians Should Know. Stat. Med..

[71] Hujoel P.P., DeRouen T.A. (1992). Validity Issues in Split-Mouth Trials. J. Clin. Periodontol..

[72] Kotsakis G.A., Javed F., Hinrichs J.E., Karoussis I.K., Romanos G.E. (2015). Impact of Cigarette Smoking on Clinical Outcomes of Periodontal Flap Surgical Procedures: A Systematic Review and Meta-Analysis. J. Periodontol..

[73] Reynolds M.A., Kao R.T., Camargo P.M., Caton J.G., Clem D.S., Fiorellini J.P., Geisinger M.L., Mills M.P., Nares S., Nevins M.L. (2015). Periodontal Regeneration–Intrabony Defects: A Consensus Report From the AAP Regeneration Workshop. J. Periodontol..

[74] Yilmaz D., Dogan N., Ozkan A., Sencimen M., Ora B.E., Mutlu I. (2014). Effect of Platelet Rich Fibrin and Beta Tricalcium Phosphate on Bone Healing. A Histological Study in Pigs. Acta Cir. Bras..

[75] Rezuc A., Saavedra C., Maass R., Poblete C., Nappe C. (2020). Histological Comparison of DBBM and Platelet Rich Fibrin for Guided Bone Regeneration in a Rabbit Model. J. Oral Biol. Craniofacial Res..

[76] Choukroun J., Diss A., Simonpieri A., Girard M.-O., Schoeffler C., Dohan S.L., Dohan A.J.J., Mouhyi J., Dohan D.M. (2006). Platelet-Rich Fibrin (PRF): A Second-Generation Platelet Concentrate. Part V: Histologic Evaluations of PRF Effects on Bone Allograft Maturation in Sinus Lift. Oral Surg. Oral Med. Oral Pathol. Oral Radiol. Endodontol..

[77] Zhang Y., Tangl S., Huber C.D., Lin Y., Qiu L., Rausch-Fan X. (2012). Effects of Choukroun’s Platelet-Rich Fibrin on Bone Regeneration in Combination with Deproteinized Bovine Bone Mineral in Maxillary Sinus Augmentation: A Histological and Histomorphometric Study. J. Cranio-Maxillofac. Surg..

[78] Tatullo M., Marrelli M., Cassetta M., Pacifici A., Stefanelli L.V., Scacco S., Dipalma G., Pacifici L., Inchingolo F. (2012). Platelet Rich Fibrin (P.R.F.) in Reconstructive Surgery of Atrophied Maxillary Bones: Clinical and Histological Evaluations. Int. J. Med. Sci..

[79] Bolukbasi N., Ersanlı S., Keklikoglu N., Basegmez C., Ozdemir T. (2015). Sinus Augmentation with Platelet-Rich Fibrin in Combination with Bovine Bone Graft Versus Bovine Bone Graft in Combination with Collagen Membrane. J. Oral Implantol..

[80] Cömert Kılıç S., Güngörmüş M., Parlak S.N. (2017). Histologic and Histomorphometric Assessment of Sinus-Floor Augmentation with Beta-Tricalcium Phosphate Alone or in Combination with Pure-Platelet-Rich Plasma or Platelet-Rich Fibrin: A Randomized Clinical Trial. Clin. Implant Dent. Relat. Res..

[81] Nizam N., Eren G., Akcalı A., Donos N. (2018). Maxillary Sinus Augmentation with Leukocyte and Platelet-Rich Fibrin and Deproteinized Bovine Bone Mineral: A Split-Mouth Histological and Histomorphometric Study. Clin. Oral Implant. Res..

[82] Pichotano E.C., Molon R.S., Souza R.V., Austin R.S., Marcantonio E., Zandim-Barcelos D.L. (2019). Evaluation of L-PRF Combined with Deproteinized Bovine Bone Mineral for Early Implant Placement after Maxillary Sinus Augmentation: A Randomized Clinical Trial. Clin. Implant Dent. Relat. Res..

[83] Pepelassi E., Kaddas C. (2020). The Use of Platelet-Rich Fibrin in Maxillary Sinus Augmentation: A Review of the Literature. J. Dent. Oral Disord..

[84] Choukroun J., Diss A., Simonpieri A., Girard M.O., Schoeffler C., Dohan S.L., Dohan A.J.J., Mouhyi J., Dohan D.M. (2006). Platelet-Rich Fibrin (PRF): A Second-Generation Platelet Concentrate. Part IV: Clinical Effects on Tissue Healing. Oral Surg. Oral Med. Oral Pathol. Oral Radiol. Endodontol..

[85] Aricioglu C., Dolanmaz D., Esen A., Isik K., Avunduk M.C. (2017). Histological Evaluation of Effectiveness of Platelet-Rich Fibrin on Healing of Sinus Membrane Perforations: A Preclinical Animal Study. J. Cranio-Maxillofac. Surg..

[86] Miron R.J., Zucchelli G., Pikos M.A., Salama M., Lee S., Guillemette V., Fujioka-Kobayashi M., Bishara M., Zhang Y., Wang H.L. (2017). Use of Platelet-Rich Fibrin in Regenerative Dentistry: A Systematic Review. Clin. Oral Investig..

[87] Liu K., Huang Z., Chen Z., Han B., Ouyang X. (2021). Treatment of Periodontal Intrabony Defects Using Bovine Porous Bone Mineral and Guided Tissue Regeneration with/without Platelet-rich Fibrin: A Randomized Controlled Clinical Trial. J. Periodontol..

