# Supplementary material for: The Adjunctive Use of Leucocyte- and Platelet-Rich Fibrin in Periodontal Endosseous and Furcation Defects: A Systematic Review and Meta-Analysis
Source: Materials (Basel). 2022 Mar 11;15(6):2088. doi: 10.3390/ma15062088 (PMC8953320; doi:10.3390/ma15062088)
Supplement: Supplementary file 1 [file materials-15-02088-s001.zip › Listings S1-S5.pdf]

Listings: Search Algorithms

Listing S1. PubMed

#1 alveolar bone loss[MeSH Terms] OR guided periodontal tissue regeneration[MeSH Terms] OR guided tissue regeneration[MeSH Terms] OR blood buffy coat[MeSH Terms] OR intrabony defects[Title/Abstract] OR endosseous defects[Title/Abstract] OR guided tissue regeneration[Title/Abstract] OR infrabony defects[Title/Abstract]

#2 graft material[Title/Abstract] OR blood buffy coat[Title/Abstract] OR platelet rich fibrin[Title/Abstract] OR L-PRF[Title/Abstract] OR T-PRF[Title/Abstract] OR A-PRF[Title/Abstract] OR leukocyte platelet rich fibrin[Title/Abstract] OR PRF[Title/Abstract]

#1 AND #2

Listing S2. Scopus

#1 "alveolar bone loss" OR "guided periodontal tissue regeneration" OR "guided tissue regeneration" OR "blood buffy coat" OR "intrabony defects" OR "endosseous defects" OR "guided tissue regeneration" OR "infrabony defects"

#2 "graft material" OR "blood buffy coat" OR "platelet rich fibrin" OR "L-PRF" OR "T-PRF" OR "A-PRF" OR "leukocyte platelet rich fibrin" OR "PRF"

#1 and #2 AND (LIMIT-TO (SUBJAREA "DENT") AND "controlled clinical trial"

Listing S3. Cochrane Library (CENTRAL)

#1 («controlled clinical trial»):pt OR («guided tissue regeneration»):ti,ab,kw OR (infrabony defects):ti,ab,kw OR (endosseous defects):ti,ab,kw OR (alveolar bone loss):ti,ab,kw (Word variations have been searched)

#2 («controlled clinical trial'):pt OR (graft material):ti,ab,kw OR (platelet-rich fibrin):ti,ab,kw

OR (leukocyte platelet-rich fibrin):ti,ab,kw OR (L-PRF):ti,ab,kw (Word variations have been searched)

#1 AND #2

Listing S4. Lilacs

#1 "alveolar bone loss" OR "guided tissue regeneration" OR "intrabony defects" OR "bone defects" OR "infrabony defects" OR "guided tissue regeneration" OR

#2 “graft material” OR “blood buffy coat” OR “leucocyte platelet-rich fibrin” OR “platelet-rich fibrin” OR “L-PRF” OR “PRF”

#1 AND #2

Listing S5. Grey Literature Report

#1 alveolar bone loss OR guided tissue regeneration OR intrabony defects OR bone defects OR infrabony defects OR guided tissue regeneration

#2 graft material OR blood buffy coat OR leucocyte platelet-rich fibrin OR platelet-rich fibrin OR L-PRF OR PRF

#1 AND #2
