# Supplementary figures and images for: The Adjunctive Use of Leucocyte- and Platelet-Rich Fibrin in Periodontal Endosseous and Furcation Defects: A Systematic Review and Meta-Analysis
Source: Materials (Basel). 2022 Mar 11;15(6):2088. doi: 10.3390/ma15062088 (PMC8953320; doi:10.3390/ma15062088)

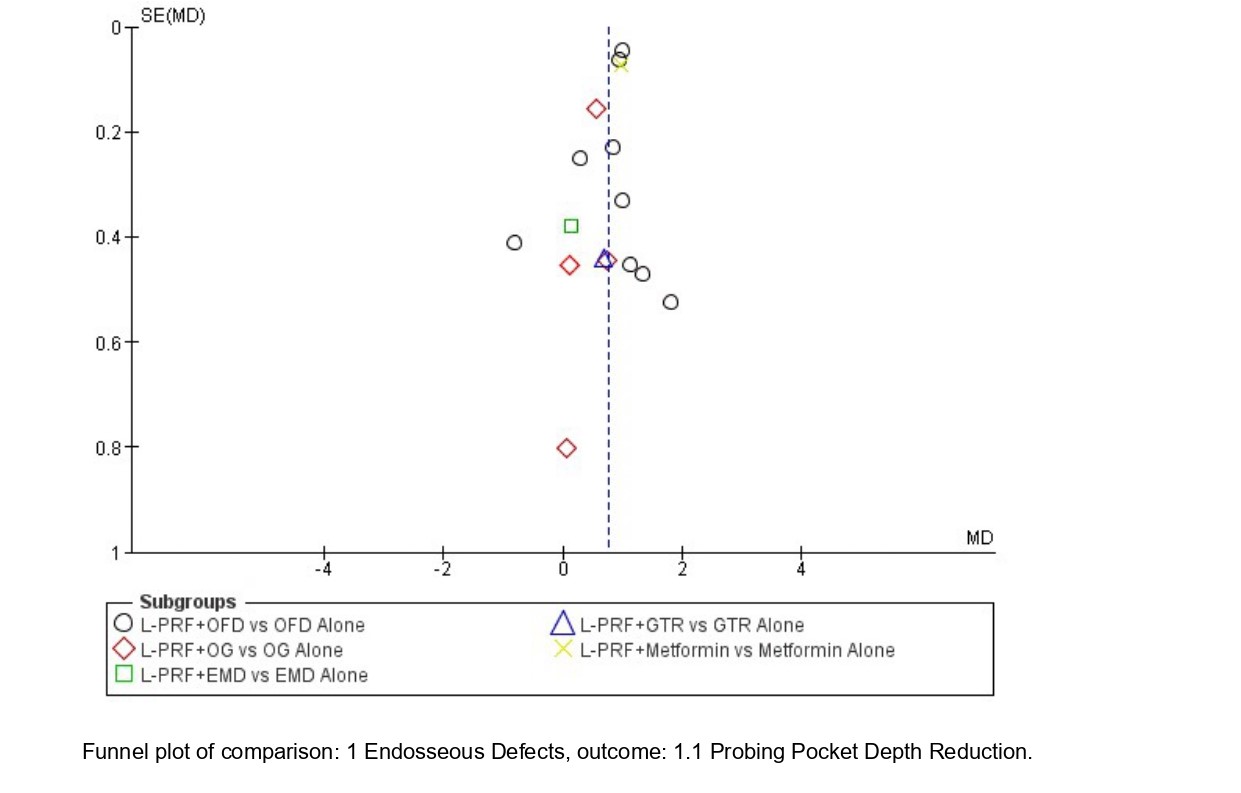

Supplement: Supplementary file 1 [file materials-15-02088-s001.zip › Figure_S1.jpg]

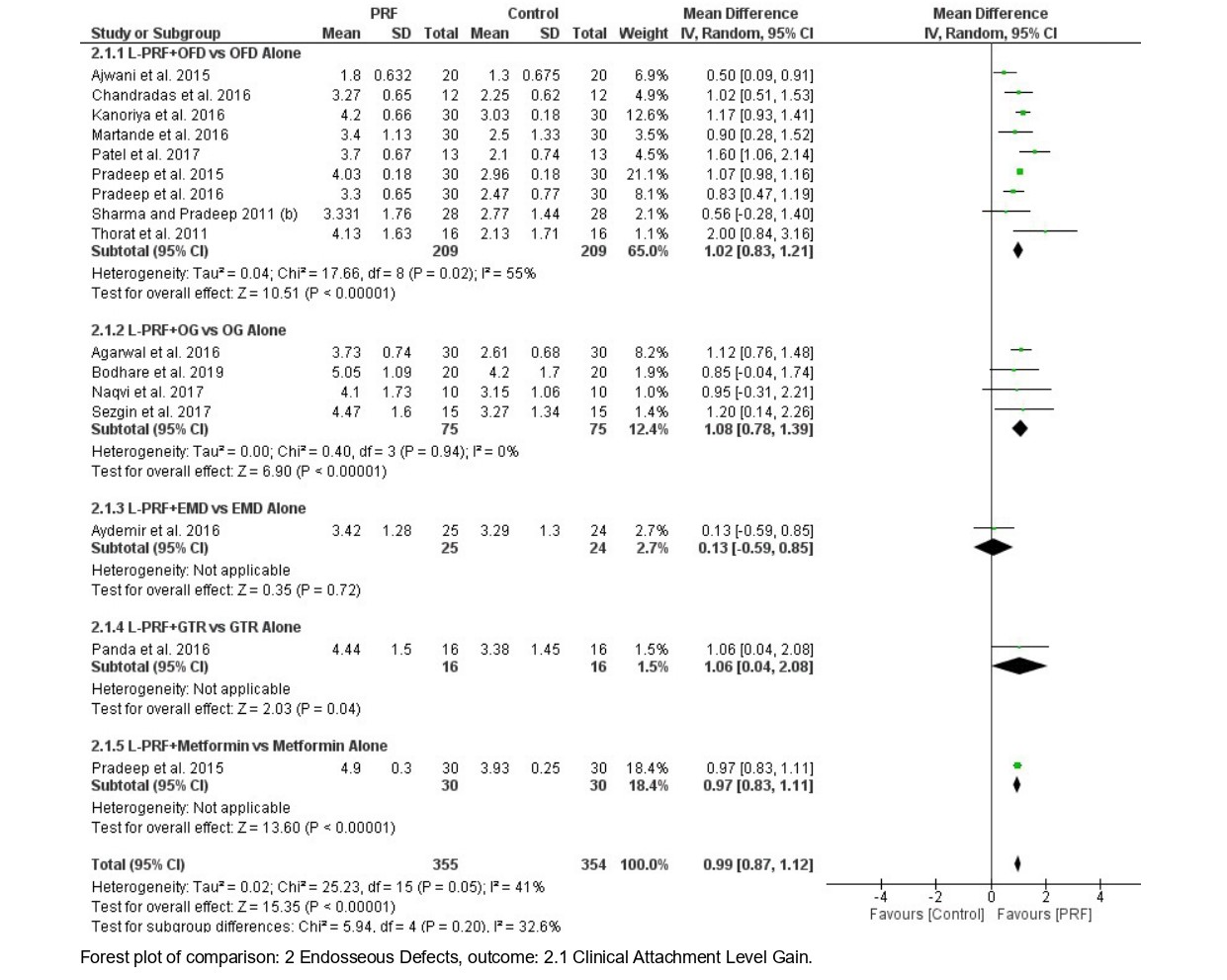

Supplement: Supplementary file 1 [file materials-15-02088-s001.zip › Figure_S2.jpg]

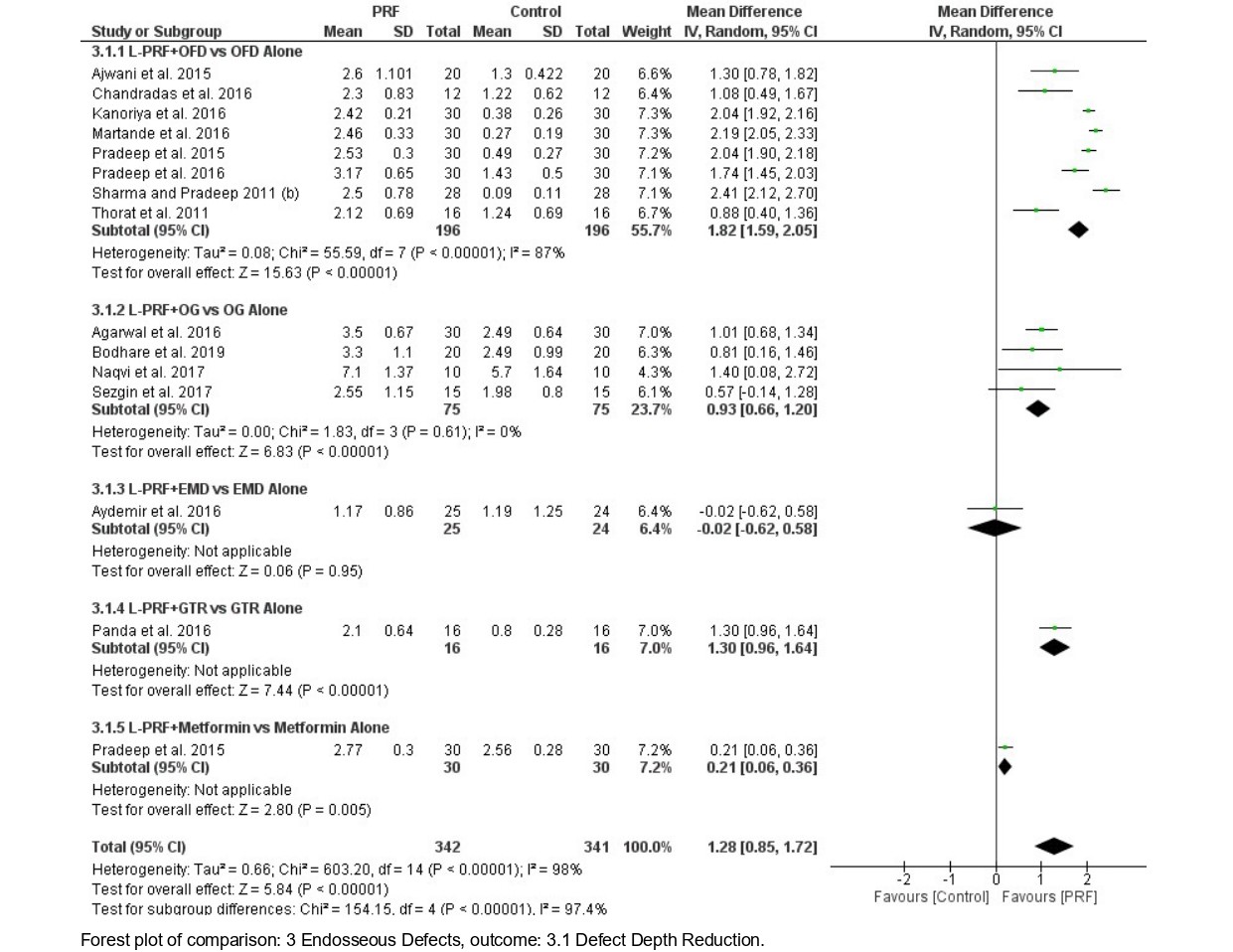

Supplement: Supplementary file 1 [file materials-15-02088-s001.zip › Figure_S3.jpg]

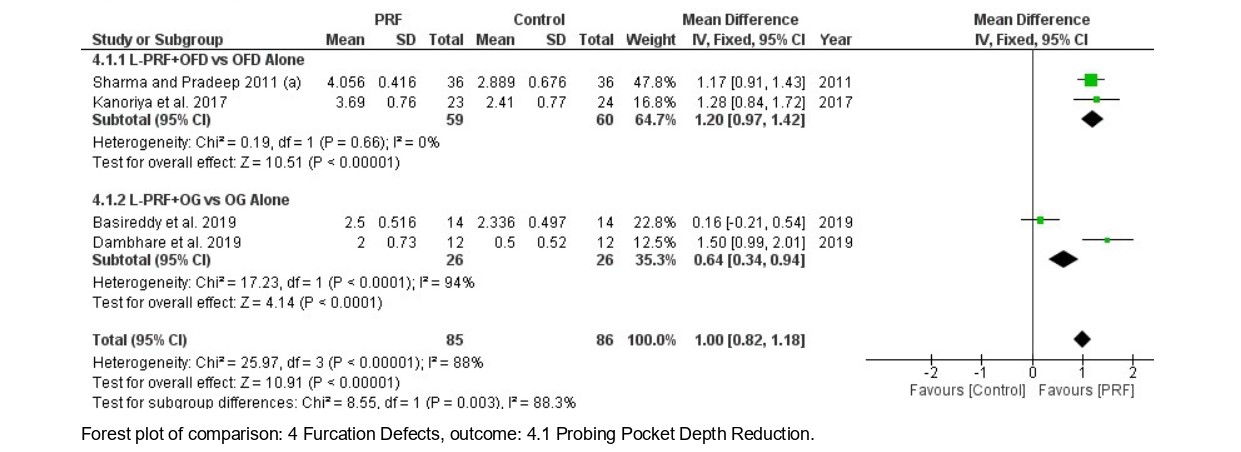

Supplement: Supplementary file 1 [file materials-15-02088-s001.zip › Figure_S4.jpg]

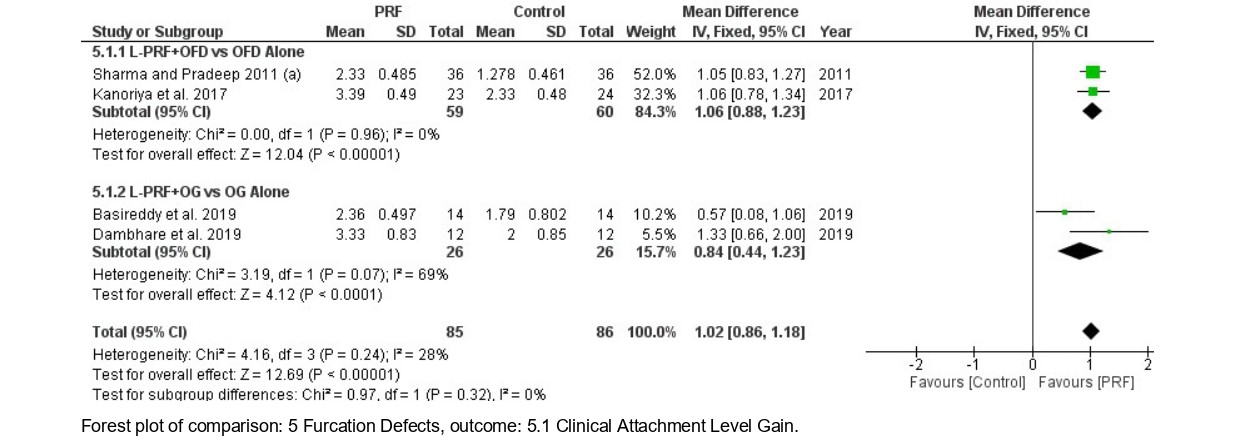

Supplement: Supplementary file 1 [file materials-15-02088-s001.zip › Figure_S5.jpg]

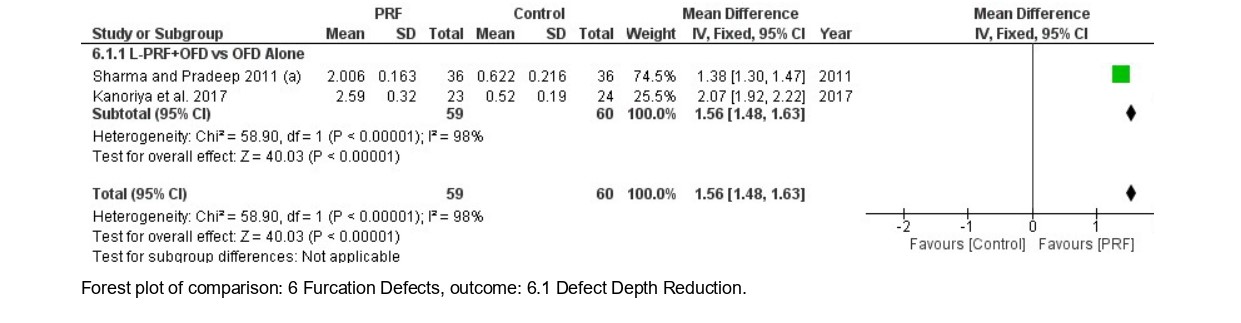

Supplement: Supplementary file 1 [file materials-15-02088-s001.zip › Figure_S6.jpg]
